# Temporal disambiguation of relative temporal expressions in clinical texts

**DOI:** 10.3389/frma.2022.1001266

**Published:** 2022-10-24

**Authors:** Amy L. Olex, Bridget T. McInnes

**Affiliations:** ^1^C. Kenneth and Diane Wright Center for Clinical and Translational Research, Virginia Commonwealth University, Richmond, VA, United States; ^2^Department of Computer Science, Virginia Commonwealth University, Richmond, VA, United States

**Keywords:** natural language processing, temporal reasoning, temporal expression recognition and normalization, clinical text, relative temporal expression, error analysis, BERT, contextual word embeddings

## Abstract

Temporal expression recognition and normalization (TERN) is the foundation for all higher-level temporal reasoning tasks in natural language processing, such as timeline extraction, so it must be performed well to limit error propagation. Achieving new heights in state-of-the-art performance for TERN in clinical texts requires knowledge of where current systems struggle. In this work, we summarize the results of a detailed error analysis for three top performing state-of-the-art TERN systems that participated in the 2012 i2b2 Clinical Temporal Relation Challenge, and compare our own home-grown system Chrono to identify specific areas in need of improvement. Performance metrics and an error analysis reveal that all systems have reduced performance in normalization of relative temporal expressions, specifically in disambiguating temporal types and in the identification of the correct anchor time. To address the issue of temporal disambiguation we developed and integrated a module into Chrono that utilizes temporally fine-tuned contextual word embeddings to disambiguate relative temporal expressions. Chrono now achieves state-of-the-art performance for temporal disambiguation of relative temporal expressions in clinical text, and is the only TERN system to output dual annotations into both TimeML and SCATE schemes.

## 1. Introduction

Temporal reasoning is a high-level natural language processing task that aims to extract and assimilate temporal information in written or spoken natural language to reconstruct a series of events such that they can be reasoned over to answer questions involving time. Temporal expression recognition and normalization (TERN) is the foundation of any temporal reasoning pipeline, so performance must be high else there is the risk of error propagation to dependent tasks, such as timeline extraction. There has been a growing amount of work done on temporal information extraction over the past several decades (Pani and Bhattacharjee, 2001; Zhou and Hripcsak, 2007; Gupta, 2015; Leeuwenberg and Moens, 2019; Lim et al., 2019; Olex and McInnes, 2021); however, performance of current state-of-the-art timeline extraction pipelines are still not good enough to integrate into clinical practice (Olex and McInnes, 2021) leaving many areas of progress open to new and innovative ideas.

Identifying accurate dates and times (i.e., temporal expression recognition and normalization) of events is especially crucial in the medical field when extracting clinical timelines for patient care. The 2012 Informatics for Integrating Biology and the Bedside (i2b2) Clinical Temporal Relations Challenge (2012 i2b2 Challenge) provided the clinical NLP community with the first temporally annotated and de-identified clinical corpus for developing temporal reasoning systems. This corpus has become a benchmark in the field of clinical temporal reasoning for defining state-of-the-art performance for tasks such as TERN; however, since this challenge there has been little progress in this area, and rule-based systems remain the preferred approach when implementing clinical NLP temporal reasoning pipelines (Olex and McInnes, 2021).

In this work, we perform a detailed error analysis of several state-of-the-art clinical TERN systems to identify specific areas in need of further improvement. While the top systems participating in the i2b2 Challenge achieved span-based F-measure scores around 0.90, indicating good performance in identifying temporal expression spans, performance metrics and an error analysis reveal that all systems have reduced performance in normalization of relative temporal expressions. Relative temporal expressions appear frequently in clinical texts, are vital to ordering events on a timeline, and are thus important to normalize correctly. This work advances progress in timeline extraction by focusing on improving the recognition and classification of relative temporal expressions in clinical texts, specifically in disambiguating temporal types, which is a required step before correct normalization can be achieved.

There are two types of Temporal Disambiguation tasks that have been historically referenced in the literature: *Temporal Sense Disambiguation* (TSD) and *Temporal Type Disambiguation* (TTD). Both are similar to the classic task of Word Sense Disambiguation (WSD) (McInnes and Stevenson, 2014; Torii et al., 2015; Antunes and Matos, 2017; Hristea and Colhon, 2020). In language, the same lexical form of a word can have multiple meanings depending on the surrounding context. For example, the word “bat” could refer to a fuzzy animal with leathery wings, or a wooden stick used to hit a ball. The WSD task is to figure out what concept the word “bat” is referring to by utilizing context clues. Similarly, the TSD task is to identify if a word, such as “spring”, is referring to the temporal sense of the Spring season, or a non-temporal sense of the word (e.g., an action or a physical spring) (Mani, 2004; Mingli et al., 2005). On the other hand, the TTD task aims to identify the temporal type of a temporal expression so that it can be normalized correctly. An example is the expression “a week ago”. In all instances, the word “week” refers to the concept of 7 days, so it has the same semantic meaning regardless of temporal type. However, TTD determines if the expression “a week ago” refer to a single point in time that an event occurred (a DATE type), or a span of time for which an event took place (a DURATION type). TTD is vital for normalizing relative temporal expressions as they have to be assigned the correct type in order to be correctly normalized and positioned on a timeline.

To address the issue of temporal disambiguation we developed and integrated a module into our TERN system Chrono (Olex et al., 2018) that utilizes temporally fine-tuned contextual word embeddings to disambiguate relative temporal expressions, and compare the performance on relative expressions of Chrono to the top i2b2 TERN systems. Chrono is freely available on GitHub.^1^

## 2. Materials and methods

Materials and Methods are organized as follows: We first summarize the i2b2 corpus (Section 2.1) and describe how the top i2b2 systems were chosen (Section 2.2). Next a description of the automated error analysis and the criteria for choosing the poorest performing files used in a detailed manual error analysis is provided (Section 2.3). Section 2.4 describes how the i2b2 data set was filtered to create new training and evaluation data sets for relative, implicit, and vague temporal expressions (RelIV-TIMEX). We then introduce our TERN system Chrono and the steps taken to modify it to output compatible annotations with the i2b2 Challenge schema (Section 2.5), followed by outlining the framework for setting up the temporal disambiguation experiments (Section 2.6). Finally, evaluation metrics are discussed (Section 2.7).

### 2.1. 2012 i2b2 clinical temporal relations challenge corpus

The 2012 i2b2 Clinical Temporal Relations Challenge corpus (i2b2 corpus) contains 310 de-identified discharge summaries from Partners Healthcare and the Beth Israel Deaconess Medical Center with gold standard temporal annotations (Sun et al., 2013). Temporal expressions are annotated with the ISO-TimeML scheme and included the types DATE, TIME, DURATION, and FREQUENCY with absolute values normalized to the ISO-8601 standard. Since the official challenge in 2012, this corpus has been used as a benchmark for evaluating progress in the temporal reasoning field (Cheng et al., 2013; D'Souza and Ng, 2014a,b; Lin et al., 2016; Wang et al., 2016; Lee et al., 2017, 2018; Patel and Tanwani, 2018; Guan et al., 2020).

### 2.2. Selecting top performers in the 2012 i2b2 challenge

The organizers of the 2012 i2b2 Challenge provide the submitted results from the top 10 participating systems with the download of the annotated data. Using the performance results from the TIMEX section of Table 2 in Sun et al. (2013), we chose to analyze the TIMEX output of the following three systems:

- Mayo Clinic: The top performing rule-based system primarily using regular expressions and built on top of HeidelTime, a top performing general domain temporal tagger (Strötgen and Gertz, 2010; Sohn et al., 2013).
- Vanderbilt: A mid-range performing, rule-based system that was also built on top of HeidelTime (Strötgen and Gertz, 2010).
- Microsoft Research Asia (MSRA): The top performing hybrid system utilizing rules, conditional random fields, and support vector machines (SVM).

### 2.3. Error analysis and choosing the poorest performing files

An error analysis was performed on all files in the evaluation set by creating a pair-wise match between the gold standard annotations and system annotations from each file using overlapping spans. Each annotation was assessed for temporal expression type labeling and value errors using an in-house Python script.^2^ These include the total number of errors of any type, number of value errors (only considering values of expressions that were labeled correctly), the total number of annotations that were mislabeled, total number that were missed (annotated by gold but not by the system), and total number that were new (annotated by the system but not by gold). For a detailed manual analysis of the most difficult phrases to correctly annotate, a subset of the poorest performing files were chosen using the i2b2 file-level evaluation results from the Mayo Clinic system. Files with any one of Precision, Recall, or Value Accuracy that was close to or less than 0.75 were chosen for analysis. Running this same selection process for the top hybrid system, MSRA, revealed no additional files that added to the types of errors being made. The resulting list contained 18 files from the i2b2 evaluation data set that were the most difficult files for rule-based and hybrid systems to parse.

### 2.4. Creating a relative temporal expression gold standard data set

For this work we utilize several variations of the 2012 i2b2 corpus for training and evaluation of relative temporal expression disambiguation. The i2b2 training data set contains 190 documents with a total of 2,366 annotated temporal expressions, and the evaluation data set contains 120 documents with a total of 1,820 temporal expressions (Table 1). For End-to-End evaluations of Chrono and the other state-of-the-art systems, as well as the training of the multi-label classification sequence-to-sequence models, the i2b2 data set is used as-is. When training classification models for the temporal disambiguation task, the i2b2 data set is filtered to only DATE and DURATION types, which is referred to as the DD-TIMEX data set, and this is further filtered to only relative, implicit, and vague temporal expressions (RelIV-TIMEX data set) for evaluation of the models. The DD-TIMEX and RelIV-TIMEX data sets are described in more detail below.

**Table 1:** Number of annotated temporal expressions for the four temporal types in the full i2b2 data set and the filtered RelIV-TIMEX data set.

**Temporal type**	**i2b2 train**	**i2b2 evaluation**	**RelIV-TIMEX evaluation**
DATE	1,641	1,222	429
DURATION	407	341	307
TIME	69	60	-
FREQUENCY	249	197	-

#### 2.4.1. Training data set: DATE/DURATION TIMEXs only (DD-TIMEX)

As this work is focused on building a classifier for the DATE/DURATION TIMEX types, the i2b2 Training and Evaluation data sets were filtered to include temporal expressions that were annotated as a DATE or DURATION only (2,047 expressions, Table 1). All TIME and FREQUENCY annotated expressions were removed from the existing gold standards. These modified data sets are used in all model training, and are referred to as the “DD-TIMEX” Training and Evaluation Gold Standards. Note that these contain relative, incomplete, vague, and absolute/explicit temporal expressions.

#### 2.4.2. Evaluation data set: RelIV-TIMEXs only

To assess the performance of the temporal disambiguation module on the RelIV-TIMEXs, all absolute/explicit or incomplete temporal expressions were removed from the DD-TIMEX Evaluation data set. Any TIMEX meeting one of the following criteria was manually removed from the DD-TIMEX Evaluation data set:

- An explicit date or time, full or partial (e.g., 2/4/2013, 9 a.m., 5/6, etc.).
- A proper month or day of the week (e.g., February, Monday, etc.).
- The name of a holiday (e.g., Halloween).

This primarily removed DATE types for a total of 429 RelIV DATEs and 307 RelIV DURATIONs (Table 1). This data set is referred to as the RelIV-TIMEX Evaluation data set and is only used for evaluation purposes.

Note that the DD-TIMEX data set is used to train all models described in Section 2.6, and the RelIV-TIMEX data set is used only for evaluation. This was done due to the limited number of relative examples so that the models would have more data to train from as the context surrounding explicit and incomplete temporal expressions can be similar to those of relative expressions. Additionally, the final model needs to be able to also classify some incomplete phrases for integration into the End-to-End pipeline.

### 2.5. Chrono overview and system modifications

Chrono is the first hybrid TERN tool that normalizes temporal expressions into the SCATE (Semantically Compositional Annotations for Temporal Expressions) Schema (Bethard and Parker, 2016), which is capable of annotating a wider range of temporal expressions than the popular ISO-TimeML schema used in the 2012 i2b2 Challenge. Chrono has evolved from parsing news wire text (Olex et al., 2018) from the AQUAINT corpus (Graff, 2002), to clinical notes (Olex et al., 2019) from the THYME (Temporal Histories of Your Medical Events) corpus (Styler et al., 2014; Olex et al., 2019), achieving state-of-the-art performance for normalization of temporal expressions into the SCATE schema. However, it has yet to be run on the benchmark 2012 i2b2 corpus as SCATE and TimeML annotations are not directly comparable. Thus, in order to compare the performance of Chrono to current state-of-the-art methods it was necessary to convert SCATE annotations to ISO-TimeML so that a direct comparison could be made.

While SCATE contains fine-grained annotations that should be easily converted to ISO-TimeML, there are some challenges in retrieving a good conversion as the SCATE XML files do not store the full temporal phrase that can contain important information for ISO-TimeML attributes; thus, any conversion script would still need access to the full text document (see Supplementary File 1 for additional details). To avoid the computational complexity of re-parsing a text file with the SCATE annotations, it was decided to integrate the needed ISO-TimeML information into the existing SCATE objects within Chrono, then provide an additional input/output mode for ISO-TimeML annotations. System modifications were implemented in two phases: (1) Conversion Changes and (2) Algorithm Improvements. Briefly, conversion changes were those modifications to convert SCATE annotations to ISO-TimeML. These included converting explicit date/time phrases into the ISO standard using the Python module “dateutil”, mapping Period and Calendar-Interval SCATE entities to a DURATION type in ISO-TimeML with the proper ISO-formatted string (e.g., “P2D” for “2 days”), and using the LSAT exam^3^ standards for setting numeric values for approximate temporal phrases such as “several days”. Algorithm improvements included changes to capture temporal elements not seen in previously used corpora (e.g. AQUAINT and THYME) (Olex et al., 2018, 2019). Using the 2012 i2b2 training data set, this phase was focused on improving Recall and included creating a clinical abbreviations dictionary and frequency method to pull in abbreviated temporal phrases such as “b.i.d.”, which represents “twice a day”. Additionally, 2-place dates, such as “01/10”, were not being identified at all, so new parsing logic was added to recognize these instances, which were ubiquitous in the i2b2 corpus. Additional details describing the changes made to Chrono can be found in Supplementary File 2.

### 2.6. Infusion of temporal information into contextualized word embeddings

Recently, there has been an increase in attention to the infusion of temporal information into contextualized embeddings with the goal of improving prediction tasks. However, the focus has primarily been on temporal relation prediction (Liu et al., 2019; Guan and Devarakonda, 2020) with some recent work on temporal tagging in the general domain (Almasian et al., 2022) and prediction of clinical outcomes (Pang et al., 2021). As of yet, there are no publications utilizing contextualized embeddings for the task of temporal disambiguation of relative temporal expressions.

**Figure 1:**
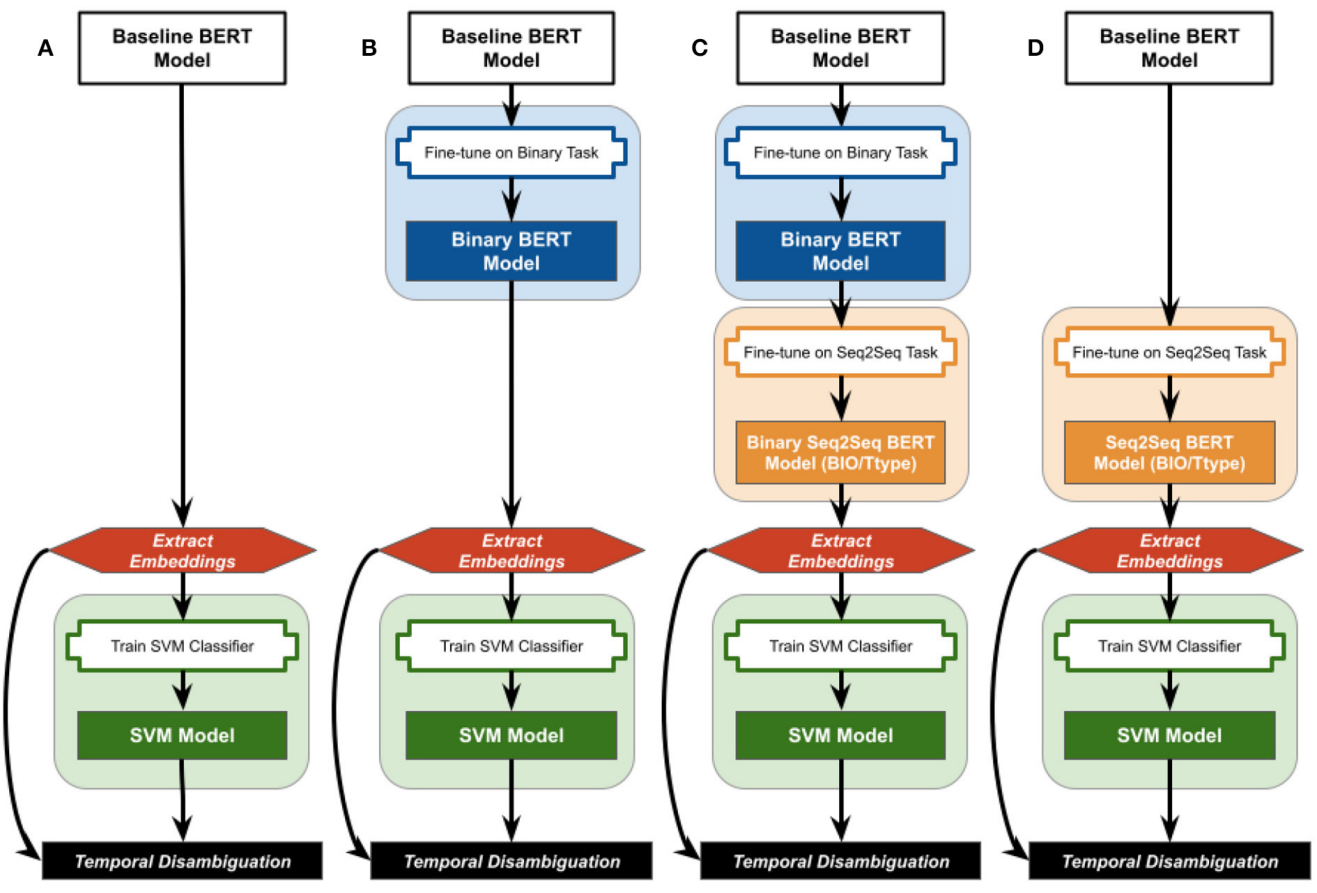
Overview of the fine-tuning, embedding extraction, and classification strategies examined in this work. Baseline BERT models are either the BertBase or ClinBioBert models referenced in the text. **(A)** No fine-tuning; **(B)** binary fine-tuning; **(C)** sequential Binary-Seq2Seq fine-tuning; **(D)** Seq2Seq fine-tuning.

This work evaluates whether fine-tuning on simplistic and/or complex temporal classification tasks embeds temporal information into the extracted contextualized embeddings from two baseline BERT (Bidirectional Encoding Representations from Transformers) models (Devlin et al., 2019). Figure 1 summarizes the various combinations of fine-tuning, embedding extraction, and classification strategies evaluated. All strategies start with either the uncased BERT Base language model (Devlin et al., 2019), referred to as “BertBase”, or the clinical BioBert model already fine-tuned on biomedical literature and clinical notes by Alsentzer et al. (Alsentzer et al., 2019), referred to as “ClinBioBert”. The strategies using the unmodified BertBase and ClinBioBert contextualized embeddings are considered the baseline for this work (Figure 1A), and are referred to as the “baseline BERT models” when discussed together.

In the following subsections, we first describe a high-level binary classification task used to fine-tune the baseline BERT models. This binary fine-tuned model is either used to obtain contextualized embeddings for input into the down-stream classification models (Figure 1B), or as the initiating model for fine-tuning sequence-to-sequence (Seq2Seq) classification models (Figure 1C). Finally, the baseline BERT models are used directly to train Seq2Seq fine-tuned BERT models, which are then used to extract contextualized embeddings for down-stream classifiers (Figure 1D).

#### 2.6.1. Fine-tuning strategies

Four strategies were employed to infuse temporal information into BERT embeddings using the 2012 i2b2 training corpus: Binary, two versions of Sequence-to-Sequence (Seq2Seq), and Sequential. Binary temporal fine-tuning was achieved by fine-tuning the existing BertBase and ClinBioBert models on the binary temporal task (Figure 1B) of classifying sentences as either containing or not containing temporal information. Configuration details, gold standard generation, and performance results for this binary classification task are detailed in Olex (2022). Briefly, the “BertForSequenceClassification” model from the HuggingFace Transformers Python library (Wolf et al., 2020) was used, and model classification performance achieved weighted F1 measures of 0.96 for both baseline BERT models.

The Seq2Seq fine-tuning task consisted of classifying each token as either non-temporal or as one of the temporal types in the ISO-TimeML schema (DATE, DURATION, TIME, FREQUENCY). Token classification was done in two ways: (1) Tokens were classified using the beginning-inside-outside (BIO) model where the “beginning” is the first token of a TIMEX, the “inside” is all subsequent tokens in a TIMEX, and any token not part of a TIMEX is labeled as “outside”. These models are referred to as Seq2Seq-BIO models. Thus, each of the four TimeML TIMEX types had two associated labels (e.g., B-date, I-date) for a total of 9 labels. (2) Tokens were classified with the temporal type only (Ttype), which are referred to as Seq2Seq-Ttype models, without differentiating between the beginning and inside of a TIMEX for a total of 5 labels. Due to the large number of “outside” labels for both the BIO and Ttype schemes, these values were excluded when calculating the evaluation metrics in order to focus on the temporal types specifically. The baseline BERT models, as well as the binary fine-tuned versions, were used as the initial models for fine-tuning each Seq2Seq classifier. Configuration details, gold standard generation, and performance results for the Seq2Seq classification task are detailed in Olex (2022). Briefly, The “BertForTokenClassification” model from the HuggingFace Transformers Python library (Wolf et al., 2020) was used. Ttype model performance for both baseline Seq2Seq models and Sequential strategies outperformed the comparable BIO model strategies with a F1 score of 0.82 for both the Seq2Seq baseline models, and a 0.61 for the sequential Binary-Seq2Seq fine-tuned versions. In addition, the Seq2Seq models for both BIO and Ttype outperformed the comparable Binary-Seq2Seq for both the BertBase and ClinBioBert baseline models (full results reported in Olex, 2022).^4^

#### 2.6.2. Feature extraction with temporally-infused contextualized embeddings

Feature extraction aims to identify a single, or set of, contextualized embeddings to be used as input for learning models. Before feature extraction, however, the embeddings returned by BERT must be pre-processed to obtain one representation per whitespace delimited token by resolving (1) subword embeddings and (2) embeddings from the multiple hidden layers of the BERT model. Briefly, we chose to use the last subword embedding to represent an entire whitespace tokenized token^5^, and the last 4 hidden layers were concatenated together to form a single contextualized embedding of length 3,072 for each word (details can be found in Olex, 2022).

After BERT embeddings were pre-processed the features used to represent a temporal phrase were constructed in one of two ways: (1) phrase only and (2) phrase plus context (Phrase+Context). Supplementary Figure 1 shows how these two strategies are implemented for the SVM architecture, which is explained in Section 2.6.3. Briefly, for the representative phrase embedding is calculated by averaging all pre-processed token embeddings that are part of a phrase. This results in each temporal phrase being represented by a single numerical vector of length 3,072 for use as a feature in the downstream classification models (Supplementary Figure 1, Phrase Only). For the Phrase+Context representation a context window of 3 words before and 3 words after the temporal phrase are taken. Each context is averaged and appended to the Phrase Only representation (Supplementary Figure 1, Phrase+Context) to obtain a single feature embedding of length 9,216. If the temporal phrase is the entire sentence, or it is located at the beginning or end of a sentence, then the temporal phrase embedding is duplicated and used as the context. Additionally, if there are less than 3, but greater than 0, tokens in either of the before/after windows, then only those tokens are utilized in the summarized context embedding, thus the window is a minimum of 1 and maximum of 3 tokens.

#### 2.6.3. Classification model

A Support Vector Machine (SVM) architecture is utilized to identify whether a temporal phrase is a DATE or DURATION temporal type using the temporally-infused contextualized embeddings previously described as features. For development and training purposes, we assume that other rules and algorithms have already identified the temporal phrase under question and have determined it to be a relative temporal expression (RelIV-TIMEX). However, before normalization can take place we need to determine its temporal type. As this is a binary choice, we chose to evaluate how a classic Support Vector Machine (SVM) model performs on this task (Olex, 2022 also reports on the performance of convolutional neural networks). The input features for the SVM model were generated as previously described from the contextualized embeddings that are sourced from the baseline BertBase or ClinBioBERT models as well as models that were fine-tuned either on the binary classification task (Binary-BertBase/ClinBioBert) or one of the Seq2Seq multi-class classifications tasks (Seq2Seq-BIO/Ttype-BertBase/ClinBioBert or Binary-Seq2Seq-BIO/Ttype-BertBase/ClinBioBert) as summarized in Figure 1.

The SVM architecture requires a single feature vector per observation (the temporal phrase) as input, and outputs a 1 or −1 as the classification. For this work, DATE is set to the positive class, and DURATION is the negative class. The DD-TIMEX Training data set was used for model training and validation (Table 1) with the RelIV-TIMEX data set used for final evaluation. Implementation details, including hyperparameter optimization can be found in Olex (2022).

### 2.7. Evaluation metrics

To assess performance of model predictions, this work reports the Precision, Recall, F1 Score, and Accuracy (Equations 1–5) using the TIMEX type annotations. The Accuracy is the class-based accuracy and is calculated from only the TIMEX annotations identified by each system that are also in the gold standard (i.e., any newly identified TIMEX phrases that are not in the gold standard are excluded from the calculation), which is consistent with the i2b2 evaluation scripts. The Precision, Recall, and F1 are calculated in two ways in this work: (1) span-based and (2) class-based. Span-based is used when determining if the TERN system identified the correct span of text. This work uses the lenient definition where any overlap in span is considered correct. All results utilize the span-based metric except for those reported in Sections 3.3.1, 3.3.2, which utilize the class-based calculations that are based off of identifying the correct temporal type for a given phrase. In most instances, the individual scores for each temporal type are summarized as a weighted average. Equation (5) shows the weighted average calculated across the DATE and DURATION temporal types, utilized in Sections 3.3.1, 3.3.2, where *s* is the metric score being averaged and *w* is the weight (i.e., number of instances for that temporal type).


(1) $$\begin{array}{l} Precision=\frac{TP}{TP+FP} \end{array}$$

(2) $$\begin{array}{l} Recall=\frac{TP}{TP+FN} \end{array}$$

(3) $$\begin{array}{l} F1=\frac{2*Precision*Recall}{Precision+Recall} \end{array}$$

(4) $$\begin{array}{l} Accuracy=\frac{TP+TN}{TP+FN+FP+TN} \end{array}$$

$$\begin{array}{l} WeightedAverage \end{array}$$

(5) $$\begin{array}{l} =\frac{\left(s_{DATE}*w_{DATE}\right)+\left(s_{DURATION}*w_{DURATION}\right)}{w_{DATE}+w_{DURATION}} \end{array}$$

## 3. Results

### 3.1. Chrono's performance improves after removing temporal adverbs

**Figure 2:**
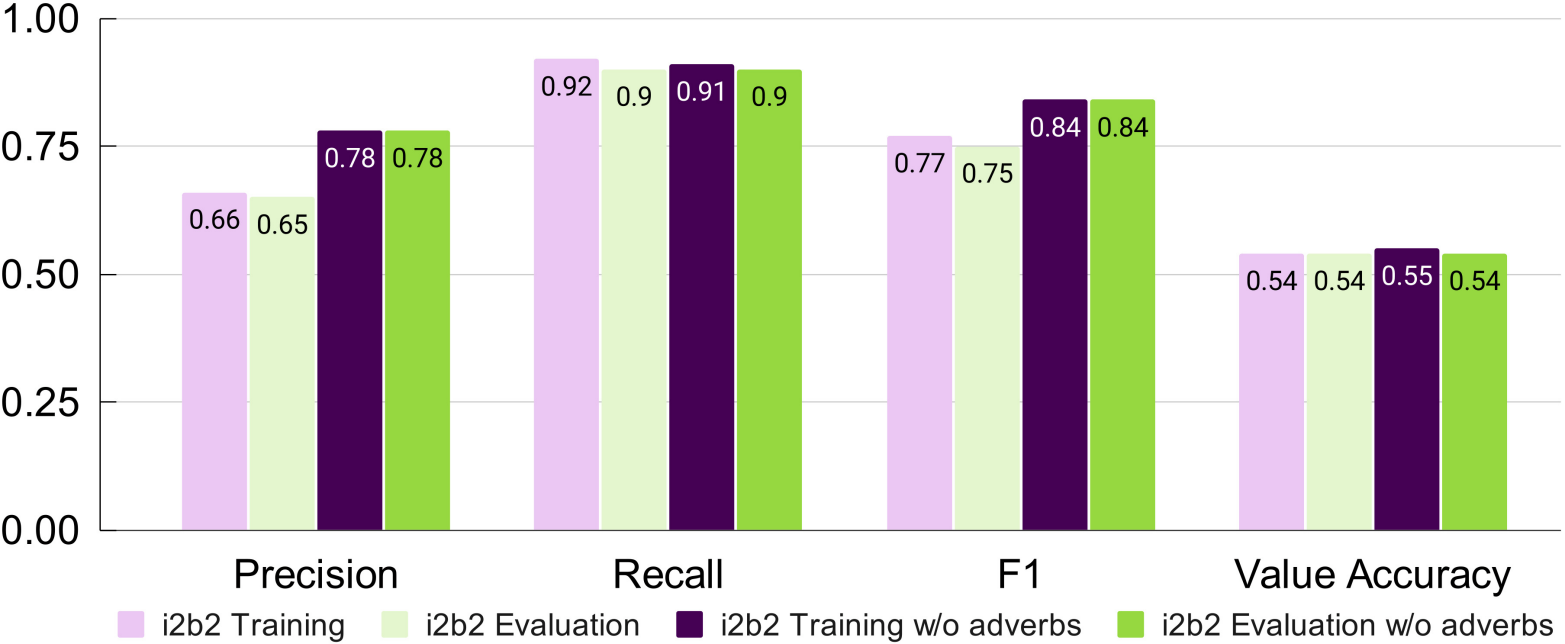
Chrono's performance on the i2b2 training and evaluation data sets after conversion changes and algorithm improvements using span-based P, R, and F1 metrics.

Chrono's performance was assessed after both phases of system modifications were implemented using the i2b2 evaluation script for the temporal expression (TIMEX) tag only. On the i2b2 training data set, Chrono achieves a Precision of 0.66, Recall of 0.92, and F1 of 0.77 (Figure 2, Light Purple). An error analysis of Chrono's performance on the i2b2 training data set revealed that the low Precision was due to Chrono annotating a lot of temporal adverbs and age-related expressions that i2b2 does not. Specifically, temporal adverbs that refer to relationships in time (e.g., “before” or “next”) or are indefinite frequencies (e.g., “often” or “rarely”) are annotated by Chrono, but ignored in the i2b2 gold standard. Removing these unannotated adverbs from Chrono's output resulted in a Precision score increase to 0.78 without affecting Recall (Figure 2, Dark Purple); thus, the “w/o adverb” settings are used for comparison to the state-of-the-art i2b2 systems in the following sections. Chrono's performance on the unseen Evaluation data set resulted in similar metrics to that obtained from the training data with just a 0.01 drop in Recall when excluding temporal adverbs (Figure 2, Green). Additional discussion can be found in Supplementary File 3.

### 3.2. Error analysis reveals six types of common errors

**Figure 3:**
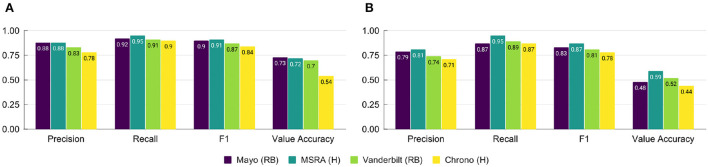
Performance of top systems from the 2012 i2b2 Temporal Challenge and Chrono on **(A)** the full evaluation data set, and **(B)** the subset of poor performing files using span-based P, R, and F1 metrics. RB, Rule-Based; H, Hybrid.

Performance on the i2b2 evaluation data set for the top i2b2 systems and Chrono is shown in Figure 3A. All systems see a large drop in performance for the Value Accuracy, indicating normalization is still a challenging task. To investigate these system errors in more detail we performed a pair-wise comparison of the gold standard annotations to those of each system using the evaluation data set. Table 2 provides the percent of temporal expression type labeling and value errors, as well as the percent of temporal expressions missed (in the gold standard but not identified by the system) and added (identified by the system, but not included in gold) by each system (error counts are provided in Supplementary Tables 1, 2). As indicated by the performance in Figure 3, errors in getting the correct value are the most frequent out of all errors. Of these value errors, 69–85% are from relative temporal expressions across the top three i2b2 systems. In contrast, Chrono has the most trouble with identifying the correct temporal type label with 43% of errors appearing in this category. Looking specifically at incorrect labels of the top i2b2 systems, 38–58% of these were due to assigning DATE instead of DURATION, or vice versa, indicating this is the most challenging labeling task for all systems, including Chrono which mislabeled 64% of these relative temporal expressions. To dive into these errors in more detail we chose 18 of the worst performing files for a more manual review. The percentage of errors for these files were similar to those calculated across all files with the exception of Mayo, which has higher error percentages as a result of the selection criteria used to pick the worst performing files (Supplementary Table 3).

**Table 2:** Percent of errors for value, label, missed, and added error categories out of all errors for the top i2b2 systems, Chrono, and Chrono plus the temporal disambiguation module (Chrono+TTD).

**System**	**Value error total %**	**Value error Rel DATE/DUR %**	**Label error total %**	**Label error Rel DATE/DUR %**	**Total % missed**	**Total % added**
Mayo	39.0	69.3	17.8	38.1	16.9	26.3
MSRA	47.0	84.9	14.7	58.7	8.9	29.4
Vanderbilt	34.0	74.1	13.6	42.5	18.9	33.6
Chrono	12.0	56.7	43.7	64.8	12.3	32.0
Chrono+TTD	42.0	72.9	11.6	29.7	12.8	33.6

Figure 3B shows the overall performance of each top i2b2 system and Chrono on the set of 18 difficult files. While Chrono performs on par with the top systems as far as Recall is concerned, Precision is about 10% lower than the others. Additionally, the type and value accuracy is lower than the top i2b2 systems as well. These metrics indicate Chrono is identifying many of the same temporal phrases as the top i2b2 systems, but work still needs to be done when assigning properties and normalized values.

Analysis of the performance of the i2b2 systems and Chrono on the selected 18 difficult files revealed several types of errors that each of the systems consistently made on the same types of temporal expressions:

- **Gold Standard:** Two of the poorest performing files were due to errors in the gold standard annotation.
- **Lexical:** Certain types of tokens were not recognized as temporal, or longer phrases were broken up so much the correct value could not be determined by the system.
- **Frequency:** Some frequencies were either missed completely or phrases were incorrectly annotated as a frequency.
- **DURATION vs. DATE:** Systems had a hard time determining if certain vague or relative temporal phrases should be annotated as a DATE type or DURATION type.
- **Anchor Time:** Systems had trouble choosing the correct anchor time to calculate dates that were referred to by relative temporal expressions.
- **Delta Values:** Errors in identifying how much time to add or subtract from an anchor time to resolve a relative temporal expression.

**Gold Standard Errors** originated from two files specifically. The first, file 208, had incomplete annotations, thus the systems were identifying temporal expressions correctly, but because these were not annotated in the gold standard the Precision suffered (around 0.3 for all systems). The second, file 83, obtained high Precision and Recall from all the systems, but very poor Value Accuracy (around 0.25). This was due to inconsistently writing the year as either 2014 or 2015 for a series of post-operative day phrases, both in the clinical note and in the annotations. If these issue had been corrected in the gold standard files, then the systems would have performed well on both files.

**Lexical Errors** included missing tokens that were annotated as temporal in the gold standard, annotating tokens as temporal that are not in the gold standard, or splitting up temporal phrases to a degree that causes incorrect value normalization. Overall, all three i2b2 systems had few lexical errors in the chosen set of files; however, the ones they did have were generally consistent across systems. For example, all systems missed annotating the phrase “three cycles” and all systems incorrectly broke up the phrase “daily for 4 days” into the phrases “daily” and “4 days” leading to incorrect temporal type assignments (Supplementary File 4 has additional examples). In comparison, Chrono fared worse than the i2b2 systems as it missed phrases such a “POD#2” that the i2b2 systems tagged. However, this is just a matter of building out Chrono's dictionary to include additional temporal phrases and abbreviations.

**Frequency Errors** included lexical issues where frequency phrases were not annotated at all, or where phrases were incorrectly flagged as a frequency. The rule-based systems from Mayo and Vanderbilt seemed to bear the brunt of these errors as their coded rules were unable to take context into account for phrases like “5 mg × 10 d” where it marked “ × 10” as a FREQUENCY, but the gold annotations and hybrid MSRA system marked “10 d” instead as a DURATION. However, the semantics of the phrase implies the patient is to take “5 mg per day for 10 days”, which includes both a FREQUENCY “per day” and a DURATION, so “correctness” here depends on how the annotations are defined. Additionally, all three systems had trouble with phrases like “negative troponin X4” where “X4” was incorrectly annotated as a frequency (Supplementary File 4 has additional examples). In contrast, Chrono's frequency module is still in its infancy where it will annotated most frequency phrase spans, but it only normalizes frequencies written as abbreviations, such as “b.i.d.”, which contributed to its poor Value Accuracy.

**Figure 4:**
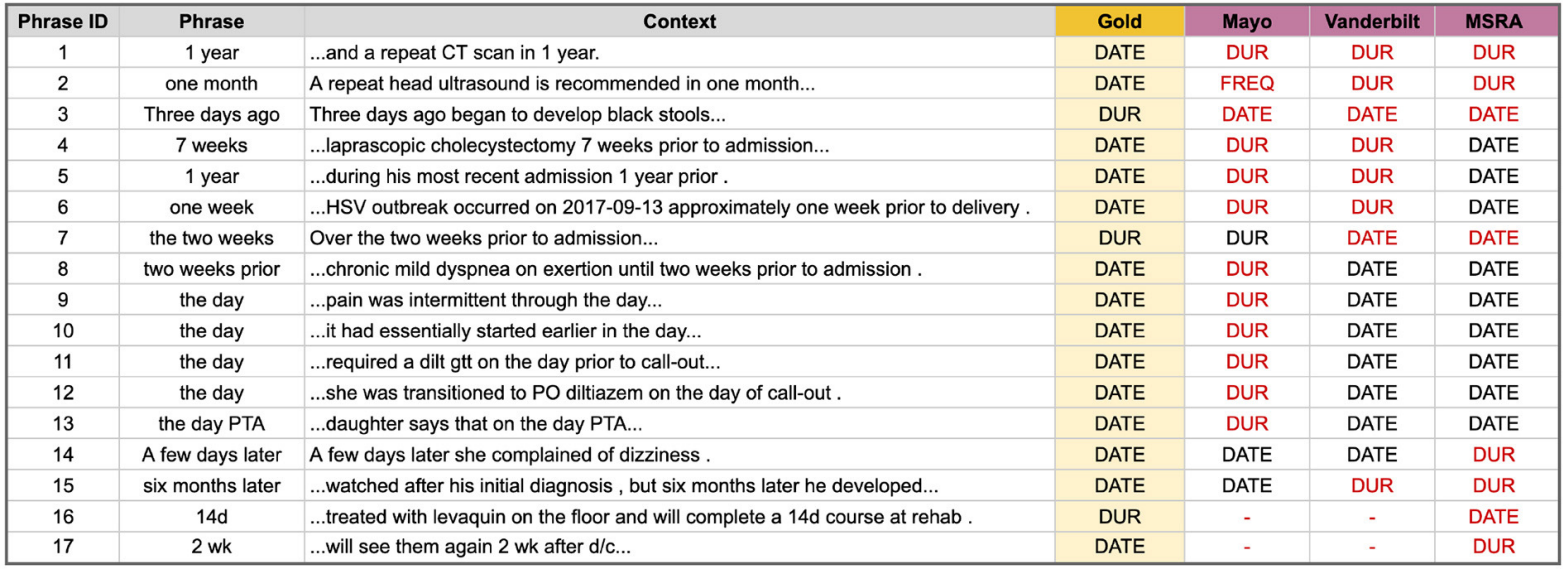
Temporal phrases that were hard to correctly classify as a DURATION or DATE temporal type. Red text indicates an incorrect classification.

**DURATION vs. DATE Errors** are those where a temporal phrase is marked as a DURATION type but should have been a DATE, or vice versa. Many DATE types are easy and straightforward to identify, such as the phrase “January 3, 2021”; however, temporal phrases that are referential or relative to an event or another time are more difficult. Figure 4 lists the 17 phrases that tripped up at least one of the top i2b2 systems. Mayo correctly classified only 3, Vanderbilt got 7, and the hybrid system from MSRA performed the best with 9 correct classifications. Note that for this type of error we are only interested in the correct Temporal Type classification and not the temporal value. The main problem areas in classifying DATE and DURATION types are (Supplementary File 4 has additional details):

- Knowing whether a related event is a discreet occurrence or something that happens continuously (Figure 4 phrases 1–3 and 16–17).
- Utilizing key context words such as “prior”, “until”, or “later” (Figure 4 phrases 4–8 and 14–15).
- Classifying the specific relative temporal expression “the day” (Figure 4 phrases 9–13).
- Knowledge of clinical shorthand (e.g., “d/c” means discharge) (Figure 4 phrases 16–17).

The two rule-based systems performed the worst on these phrases, possibly due to their static rules that may not take all context into account, or which assigns the same priority to one temporal type over another in all situations (e.g., DURATION seems to be prioritized over DATE in the Mayo system). While it is unknown if the MSRA system actually used a machine learning model for this task (no system description could be found), it is clear that this system did perform better than either of the two rule-based systems on these difficult phrases. Developing an exhaustive set of rules to identify any DURATION or DATE in any context may be infeasible due to the variety of potential lexical and semantic forms; however, a machine learning model may be able to pull this off with the right features.

For Chrono, the DURATION vs. DATE errors were the second most problematic, and were frequently tied to lexical parsing issues as well as hard-coded rules. Prior to parsing temporal phrases into the TimeML schema, Chrono first parses text into the SCATE schema. In this schema phrases such as “day of life X” only have the “day” token parsed. So Chrono is recognizing part of this phrase, hence the high recall, but it is setting it to a “Period” type. Currently, any Period or Calendar-Interval types in the SCATE schema are automatically set to a DURATION type in TimeML as that was the most frequent association, thus, regardless of the context, these types of phrases will be set incorrectly to a DURATION instead of a DATE.

**Figure 5:**
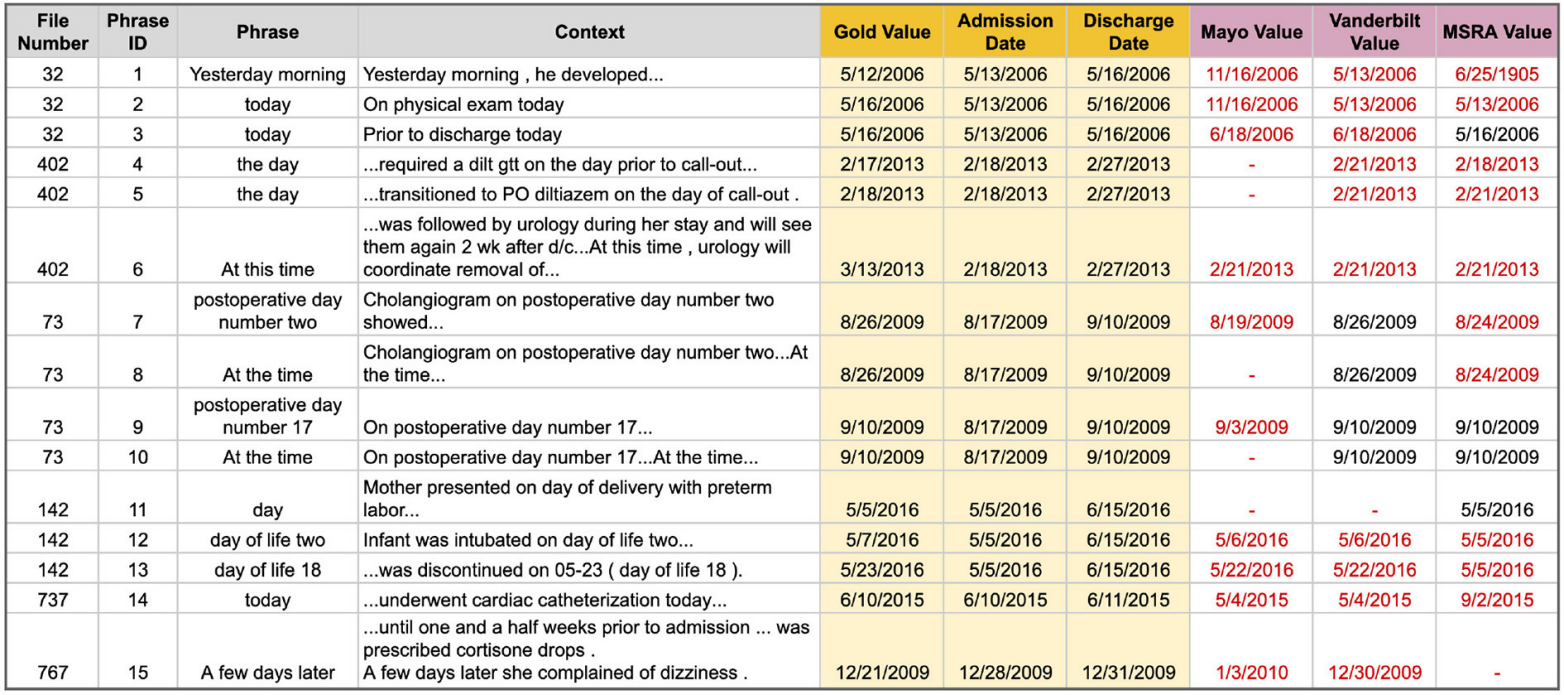
Temporal phrases for which it was hard to correctly identify the Anchor Time and/or Delta Value. Red text indicates an incorrect date.

**Anchor Time** and **Delta Value** errors go hand in hand, so will be discussed jointly. An *Anchor Time* is a calendar date used as the starting point for calculating the actual date a relative, vague, or implicit temporal phrase is referring to. For example, in the phrase “2 weeks prior to admission” the Anchor Time would be the date of admission. To calculate the calendar date being referred to in this phrase you would also need to identify the value (i.e., how many days) to add or subtract from the Anchor Time, which we refer to as the *delta value*. Anchor Times and Delta Values are only needed for relative, vague, or implicit temporal phrases classified as a DATE type in the TimeML schema. These errors were the most pervasive throughout all poor performing files and across all systems, making Anchor Time and Delta Value errors the top cause of poor performance. Figure 5 lists several example phrases, out of a total of 49, that had an Anchor Time or Delta Value error by at least one system (the full table, along with more detailed discussion, can be found in Supplementary File 4 and Supplementary Table 4). Mayo got 2 dates correct, MSRA got 8 correct, and Vanderbilt performed the best by identifying 11 dates correctly, primarily due to a single file. As the majority of these phrases were classified as a DURATION in Chrono, it is not possible to directly compare performance on this aspect to the top i2b2 systems.

There are 5 main areas of difficulty associated with Anchor Times and Delta Values:

- Temporal context switching when notes were written on multiple days.
- Unable to decipher whether the admission or discharge date is the anchor time.
- Notes referencing multiple days of care as “postoperative day” or “day of life”.
- Incorrectly using the last annotated date as the anchor time.
- Upstream annotation errors leading to a cascade of downstream errors.

The most complex challenge is determining temporal context switches to figure out what date or event a relative temporal phrase is referring to, especially in documents that were written over multiple days and don't necessarily specify which day each section was written. For example, phrases 1, 2, and 3 in Figure 5 include the temporal words “yesterday” and “today” in the same file, however, one has the admission time as an anchor while the other refers to the date of discharge. The phrase “Yesterday morning” was included in the “HISTORY AND REASON FOR HOSPITALIZATION” section of the note, while the phrase “today” was in the “HOSPITAL COURSE” section, indicating that the section location of a phrase could be important in identifying the anchor time and context switches.

Utilizing sections of a clinical note will help for some phrases, but may not always, such as for phrases 4–6, which provide examples from a single file and single notes section that reference the day of admission, the day prior to admission, and a day 2 weeks after discharge. Reasons for missing these phrases varied widely per system. Mayo annotates 2 of these 3 phrases as a DURATION instead of a DATE; for phrase 4, MSRA misses the key word “prior” and incorrectly assigns this phrase as the day of admission; and Vanderbilt uses the last annotated date from several sentences prior in the phrase “...was weaned off her pressors on 02-21...” as the anchor date. Phrases 5 and 6 are then the result of cascading errors for both Vanderbilt and MSRA. The most difficult phrase is 6, which requires context from further away (i.e., “2wk after d/c”) and over multiple sentences to normalize correctly. Unfortunately, none of the systems were able to correctly annotated the key phrase “2wk after d/c”, which would have given the correct Anchor Time for phrase 6.

Another challenge is when notes use relative expressions to reference multiple days of care, as well as using relative phrases that refer to events that happened on specific days of care. Phrases 7 through 10 in Figure 5 show a few examples from a single file that tripped up two of the top i2b2 systems. The Mayo system incorrectly assumed the anchor time was the date of admission; however, the key phrase “the patient was taken to the Operating Room on 2009-08-24” should have been used to set the anchor time for all the subsequent postoperative phrases. While the Mayo system incorrectly set the Anchor Time, it was able to correctly identify the Delta Values to calculate the date of “postoperative day” phrases, they were just consistently off by a few days, which led to poor performance on this file. The MSRA system also performed poorly on this file, however, it had the opposite problem from Mayo. MSRA was also able to ascertain the correct anchor date, but it was unable to process the delta value correctly when they were spelled out (“three” vs. “3”), which resulted in most of the postoperative phrases being set to the day of the surgery (note phrase 9 was calculated correctly). Finally, all systems were able to assign the “correct” values to the various “at this time” phrases, as these phrase values match the postoperative day dates assigned in the previous sentence (phrases 8 and 10). The performance of each system on this file indicates the importance of not just assuming an operation or other medical event happened on the day of admission and instead looking for contextual clues as to what the anchor time should be for each phrase. This, however, can be very challenging, even for a human. For example, in phrases 11-13 the “day of life X” phrases are interpreted to be from the admission date; however, the context of phrase 13 includes a specified date: “...was discontinued on 05-23 (day of life 18)”. Using this information to back-calculate when day of life 1 was we end up with the anchor date being the day after admission; thus both the Mayo and Vanderbilt systems were all off by a single day for this set of phrases. Notably, identifying this particular anchor date is a very complex task and requires high-level reasoning; thus, while assuming the day of delivery/operation is the admission date probably catches many of these instances, it is not always going to work as context needs to be considered.

A common error all three top systems made was assuming the last annotated date was in the same temporal context as the current relative temporal expression, and then using that date as the Anchor Time. For example, in phrase 14 “...underwent cardiac catheterization today...”, the term “today” was annotated by all three systems, but the date was calculated incorrectly because all three systems used some other previously annotated date as the anchor instead of setting “today” as the date of admission, which is what was annotated in the gold standard. Thus, having a blanket rule to classify these referential dates as the last annotated will certainly catch some, but will not be very precise as seen here and in additional examples discussed in Supplementary File 4.

Finally, phrases 6 and 15 show examples of how prior incorrect annotations can have downstream effects on whether or not certain referential phrases can be calculated correctly. These phrases show a chain of referring temporal phrases, thus, if the first phrase in the chain is calculated incorrectly, or not annotated at all, that affects the downstream interpretations as well, leading to cascading errors.

### 3.3. SVM model results

Three evaluation phases (Table 3) were implemented to identify the best performing SVM model for temporal type classification of relative expressions. First, the temporal phrases defined by the annotations in the RelIV-TIMEX Gold Standard Evaluation data set (Section 2.4.2) are used to build the features, i.e., no temporal phrase recognition is performed. Second, the classification models from the top performing feature extraction strategy plus the baseline BERT models are integrated into Chrono and evaluated using Chrono's temporal phrase recognition algorithm against the RelIV-TIMEX Gold Standard. These results are then compared to the performance of the top i2b2 systems for RelIV-TIMEXs only. Finally, End-2-End evaluation is performed using the best strategy implemented in Chrono and compared to the top i2b2 system results on the complete i2b2 Evaluation data set. For Phase 1 and 2 evaluations the metrics Precision, Recall, F1 and Accuracy are calculated using the TIMEX type classification (i.e., DATE or DURATION) to evaluate performance on a specific data set (see Equations 1–5), and Phase 3 evaluations use the span-based versions of Precision, Recall and F1. The weighted average (Equation 5) uses the system-specific number of DATE or DURATION instances as the weights for each metric across the DATE and DURATION results, and is used for system ranking. In the following subsections, the results of each evaluation phase are provided with discussion. All performance scores and confusion matrices for each model can be found in the Appendix of Olex (2022).

**Table 3:** Evaluation phases for classification models.

**Phase**	**Evaluation data set**	**Included systems**	**Description**
Phase 1: Gold TIMEXs	RelIV-TIMEX	Chrono	Relative temporal expression spans annotated by the i2b2 gold standard are used for temporal disambiguation.
Phase 2: System TIMEXs	RelIV-TIMEX	Chrono, Mayo, Vanderbilt, MSRA	Relative temporal expression spans annotated by Chrono and the top i2b2 systems are used for temporal disambiguation.
Phase 3: End-2-End	Complete i2b2	Chrono, Mayo, Vanderbilt, MSRA	Complete results, including all temporal expressions and types, annotated by Chrono with the temporal disambiguation module and the top i2b2 systems are evaluated.

#### 3.3.1. Evaluation phase 1: Using RelIV-TIMEX gold standard temporal phrases

**Figure 6:**
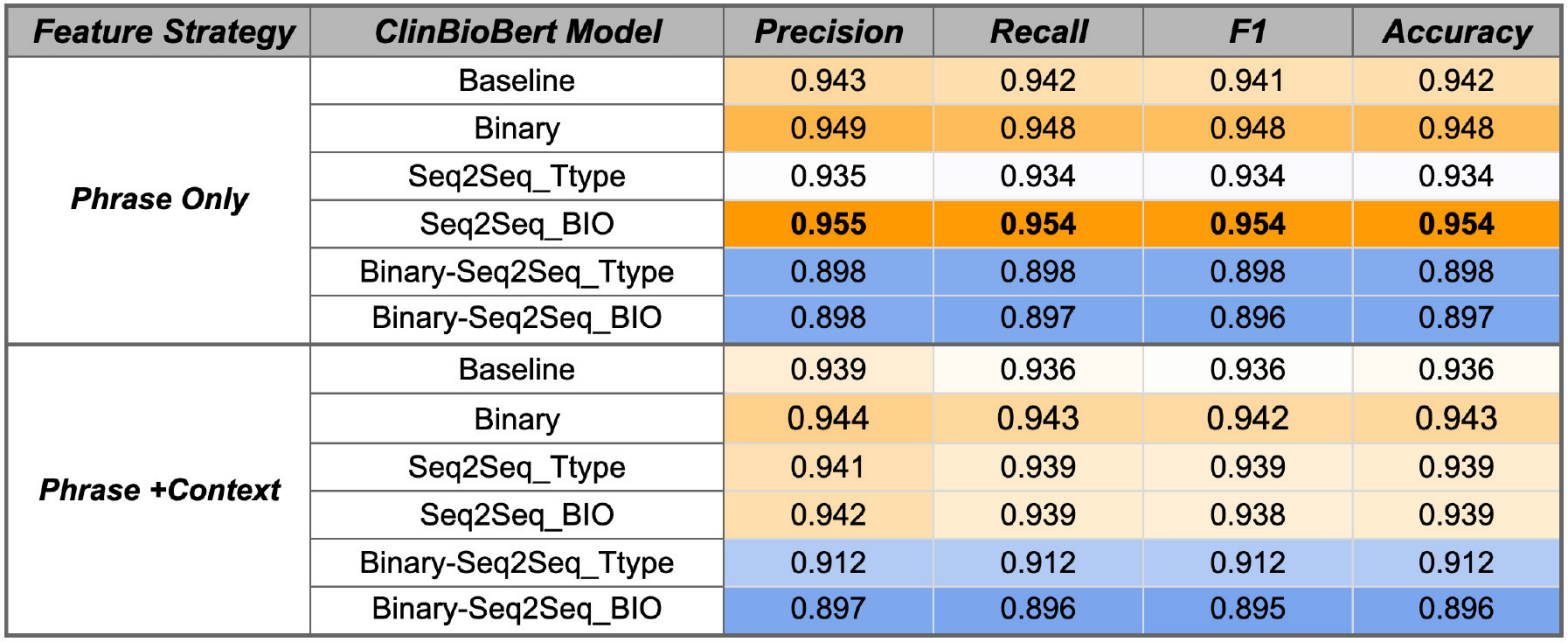
ClinBioBert SVM performance using the Gold Standard RelIV-TIMEX Evaluation data set using class-based P, R, and F1 metrics. Scores are weighted averages across DATE and DURATION. Bold, best performance across all SVM models; orange, high; white, median; blue, low scores relative to all scores in the table.

**Figure 7:**
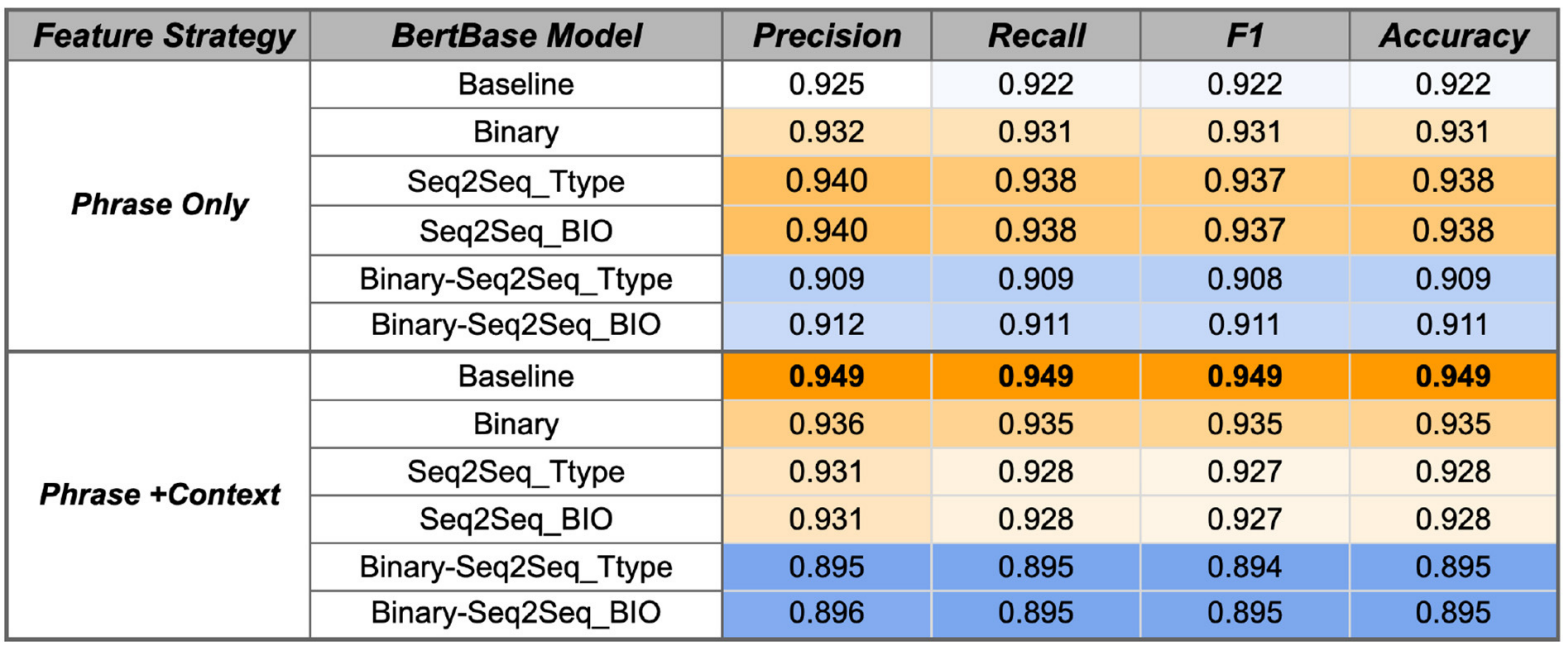
BertBase SVM performance using the Gold Standard RelIV-TIMEX Evaluation data set using class-based P, R, and F1 metrics. Scores are weighted averages across DATE and DURATION. Bold, best performance across all SVM models; Orange, high; white, median; blue, low scores relative to all scores in the table.

The SVM model variations were first evaluated using the temporal phrases from the RelIV-TIMEX Gold Standard evaluation data set as input, which contains 429 DATE types and 307 DURATION types (Section 2.4.2). The SVM results for baseline BERT model variants are shown in Figures 6, 7. Main findings and conclusions are discussed below.

##### 3.3.1.1. Temporal fine-tuning on a single temporal task improves performance

Fine-tuning the ClinBioBert model on either the binary temporal sentence classification task or the multi-label Seq2Seq temporal type classification improves classification performance from the respective baseline models (Figure 6). Specifically, the ClinBioBert-Seq2Seq-BIO fine tuning strategy (Figure 1D) achieves the highest F1 results with a score of 0.954. In contrast, sequentially fine-tuning on the binary task followed by the Seq2Seq task (Figure 1C) results in a substantial degradation of performance with the majority of F1 scores being at or less than 0.91. The observation of degraded performance after sequential fine-tuning holds true for both feature selection strategies (Phrase Only, Phrase+Context) and baseline BERT models (Figures 6, 7).

For both ClinBioBert and BertBase baseline models, fine tuning on a more complex temporal task (Seq2Seq temporal type classification) vs. a more simplistic task (binary temporal sentence classification) generally results in better performance for the Phrase Only feature selection strategy; however, the inverse is seen for the Phrase+Context strategy where the simpler binary fine tuning task results in features that outperform both Seq2Seq strategies. These results indicate that fine-tuning on a single temporal task creates contextualized embeddings that are more relevant to the temporal disambiguation task compared to those extracted from the baseline BERT models except for the BertBase baseline when using the Phrase+Context feature extraction strategy, which obtains good performance without any additional fine-tuning.

##### 3.3.1.2. Adding context helps Bertbase embeddings compensate for domain shifts

As discussed, additional fine-tuning on a single temporal task improves performance for the ClinBioBert models and the BertBase Phrase Only feature selection strategy. However, the inverse is true for the Phrase+Context BertBase models where any type of fine tuning degrades performance from the baseline model (Figure 7). Overall, the BertBase Phrase+Context SVM classifier is the highest F1 out of all combinations (Figure 7) with an F1 score of 0.949, which is not far behind the best ClinBioBert SVM classifier with an F1 of 0.954. This could be the result of the ClinBioBert models already containing the needed context in the embeddings as this model was essentially created from chaining fine-tuning tasks on biomedical and clinical texts. Thus, incorporating context explicitly may be adding too much noise. However, the BertBase model has no clinical or biomedical information already embedded; thus, explicitly including context into the extracted features from the unmodified BertBase embeddings seems to help it compensate for a domain shift.

##### 3.3.1.3. Best strategy and model

Overall, the best performing classifier is the SVM using the ClinBioBert Seq2Seq BIO model using the Phrase Only strategy with an F1 score of 0.954. The ClinBioBert and BertBase Seq2Seq Ttype and binary fine-tuned models also performed well; thus, these 6 models plus the respective baselines using the Phrase Only and Phrase+Context feature extraction strategies were moved forward to the next phase of evaluation that includes integration with the Chrono temporal phrase recognition algorithm and comparison to state-of-the-art systems that participated in the 2012 i2b2 Challenge.

#### 3.3.2. Evaluation phase 2: Integration of the temporal disambiguation module into chrono

The ClinBioBert-Seq2Seq-BIO SVM model was found to perform the best when using the gold standard temporal phrases; however, temporal phrase recognition algorithms do not always identify the exact phrase annotated in a gold standard. Thus, the next evaluation phase integrated the temporal disambiguation model into Chrono to utilize Chrono's temporal phrase recognition algorithm. Supplementary Figure 2 is a an overview of Chrono's architecture with the temporal disambiguation module shown in the workflow. Specifically, Chrono identifies and classifies temporal phrases using the fine-grained SCATE Schema. It then converts these SCATE annotations into TimeML formatted annotations. If an entity is identified as a Period or Calendar-Interval during the conversion process, it is sent to the Temporal Disambiguation module where it is classified as a DATE or DURATION type. Depending on the temporal type identified, the phrase is then sent to the TimeML Normalization module before being output to an XML file.

In this phase the performance of Chrono is still being compared to the RelIV-TIMEX Gold Standard. Since Chrono identifies all temporal expression types, the results have to be filtered to only those that overlap the RelIV-TIMEX Gold Standard. The following sub sections discuss how results from Chrono and the three state-of-the-art i2b2 systems were filtered to obtain a fair comparison to the RelIV-TIMEX Gold Standard. Then the performance of Chrono using the temporal disambiguation module is reported along with a comparison to the state-of-the-art RelIV-TIMEX performance.

##### 3.3.2.1. Creating a fair comparison to the RelIV-TIMEX data set

Previously, the temporal disambiguation module was evaluated only on RelIV-TIMEXs in the RelIV-TIMEX data set. In order to have a fair comparison, a Python script was written to filter Chrono and state-of-the-art system results to only those that overlapped with a temporal phrase in the RelIV-TIMEX Evaluation data set.^6^ This resulted in a varying number of DATE and DURATION types for each system due to the systems breaking up the gold standard phrases into multiple phrases. For example, the gold standard phrases “the morning on the day” and “hospital day #2 through hospital day #3” were generally broken up into multiple phrases by one or more of the systems. The total resulting DATE and DURATION phrase numbers are listed in Supplementary Table 5.

##### 3.3.2.2. Improved performance with temporal disambiguation module

**Figure 8:**
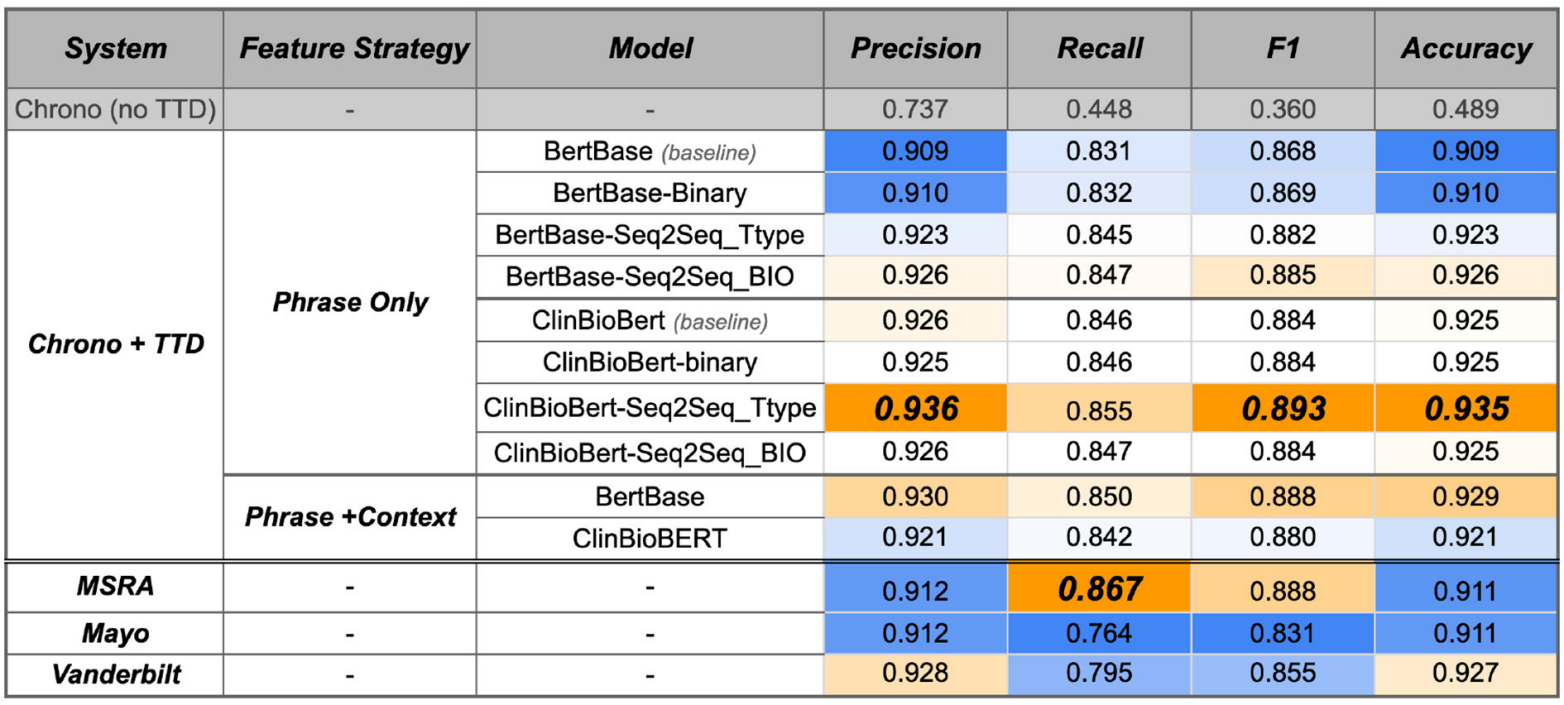
System performance on the RelIV-TIMEX Evaluation data set of Chrono before and after the TTD model integration, and the three i2b2 state-of-the-art system using class-based P, R, and F1 metrics. Values are the weighted average bootstrap estimates across individual DATE and DURATION performance (Supplementary Table 5 contains the 95% confidence intervals). Bold, best performance across all SVM models; orange, high; white, median; blue, low scores with the maximum and minimum relative to each column instead of the entire table.

Integrating any of the temporal disambiguation models from the previous section into Chrono results in a performance improvement (Figure 8, top row vs. “Chrono+TTD”; Supplementary Table 6 contains 95% confidence intervals from a bootstrap analysis of this evaluation of this phase). Previously, Chrono had a naive rule that assigned all SCATE Period and Calendar-Interval types to a TimeML DURATION type. This resulted in poor performance with a weighted F1 value of 0.360. As expected, all +TTD (Temporal Type Disambiguation) variations improve this baseline performance. The ClinBioBert models, overall, performed better than the BertBase models with the ClinBioBert Seq2Seq Ttype model achieving the best F1 score of 0.893 on the RelIV-TIMEX Evaluation data set. Interestingly, when using BertBase as the initiation model, fine-tuning on progressively more complex tasks (i.e., binary to Seq2Seq-Ttype to Seq2Seq-BIO) also continually improved performance over the baseline model. This same observation does not hold when using the ClinBioBert model as the initial model as the binary and Seq2Seq-BIO fine-tuning performed similarly to baseline while the Seq2Seq-Ttype fine-tuning resulted in the top performing model with a weighted F1 score of 0.893 (Figure 8).

All of the models just discussed utilized the Phrase Only feature strategy because it was observed that adding in context terms generally degraded performance. When adding context, a similar degradation of performance is observed. This was the case for all except the BertBase model where adding context improved performance significantly. Thus, the baseline models plus context were run with Chrono to see if the improved BertBase performance held. Indeed, the BertBase+Context model, without any fine tuning, actually achieves the second highest performance with an F1 score of 0.888. Curiously, the same strategy of adding context to the ClinBioBert baseline model actually degrades the performance compared to the Phrase Only feature strategy with an F1 of 0.880 vs. 0.884, respectively. The Seq2Seq BIO module increased the weighted F1 score to 0.884. Surprisingly, while the Seq2Seq-BIO model outperformed Seq2Seq Ttype on the RelIV-TIMEX Gold Standard phrases, the Seq2Seq Ttype model performs the best when integrated into Chrono.

##### 3.3.2.3. Chrono achieves state-of-the-art performance on relative temporal expression disambiguation

While it is good to know performance has improved with the new temporal disambiguation module, its performance needs to be compared with the other state-of-the-art systems on the same data set. For a fair comparison, the same filtering script was used on the state-of-the-art system results to obtain only those that overlap with the RelIV-TIMEX Evaluation data set. The bottom three rows of Figure 8 contain the results of the RelIV-TIMEX evaluation on the state-of-the-art systems, and Supplementary Table 6 contains the 95% confidence intervals from the bootstrap analysis. Except for Recall, Chrono plus the ClinBioBert-Seq2Seq-Ttype module achieves the highest performance for all other metrics, including the best F1 score of 0.893 compared to the top state-of-the-art system, MSRA, with an F1 of 0.888. The MSRA system achieves the highest Recall of 0.867, however, this is offset by a lower Precision of 0.912 compared to Chrono's Precision of 0.936. Additionally, all of the ClinBioBert models exceed the Mayo and Vanderbilt performances for the majority of the metrics, with the BertBase models seeing higher Precision after fine tuning.

Comparing the confusion matrices of Chrono's best performing model and MSRA, which is also a hybrid system, reveals that Chrono is better at classifying DURATION type phrases than MSRA. Chrono has a low misclassification of only 19 phrases (Table 4) compared to MSRAs 48 (Table 5). Additionally, the confusion matrices and the overall Recall score show that MSRA is identifying more relative temporal phrases overall with 38 “na” values vs. Chrono's 68. This, however, is a function of Chrono's temporal phrase recognition algorithm, which isn't affected by the TTD module. Thus, improvement in Chrono's recognition algorithm should increase performance even further.

**Table 4:** Confusion matrix for Chrono+TTD (ClinBioBert-Seq2Seq-Ttype) using the RelIV-TIMEX Evaluation data set.

		**Chrono+TTD**		
		**DATE**	**DURATION**	**na**
Gold	**DATE**	383	29	48
	**DURATION**	19	298	20

**Table 5:** Confusion matrix for MSRA using the RelIV-TIMEX Evaluation data set.

		**MSRA System**		
		**DATE**	**DURATION**	**na**
Gold	**DATE**	413	20	21
	**DURATION**	48	272	17

The same error analysis from Section 2.2 was performed on Chrono's results with and without the best performing temporal disambiguation module (ClinBioBert-Seq2Seq-Ttype). Prior to implementing the TTD module, 43% of all errors were due to labeling errors with about 64% of those directly due to labeling relative temporal expressions as a DATE or DURATION. This is in comparison to the state-of-the-art systems having an average of 15% total labeling errors with about 46% of those due to labeling relative temporal expressions as a DATE or DURATION. After implementing the TTD module, Chrono's percentage of labeling errors across all files is now down to 11% with 29% of those due to labeling DATE/DURATION relative expressions, both of which are lower rates than all state-of-the-art systems, and this performance hold for the 18 poorest performing files as well (Supplementary Table 3). As a results of improving labeling errors for relative expressions the number of value errors have increased. This is due to Chrono not yet implementing a strategy to address the Anchor Time and Delta Value issues discussed in Section 2.2, which is considered future work.

#### 3.3.3. Evaluation phase 3: End-2-End performance evaluation

The final phase of evaluation is to incorporate the best performing temporal disambiguation module into Chrono and evaluate the performance on the full set of returned annotations, i.e., End-2-End evaluation. For the End-2-End evaluation, the i2b2 evaluation scripts using span-based metrics were used unmodified. Note that Chrono's performance for the span-based Precision, Recall, and F1 scores does not change as the temporal disambiguation module only classifies a temporal expression. The goal of this phase is to see an improvement in the “Type Accuracy”. With this in mind, the Value and Modifier metrics will change, however, optimizing these is future work as no changes were made to the normalization module in Chrono. Table 6 shows the final End-2-End results using the best performing temporal disambiguation module from the previous section, ClinBioBert-Seq2Seq-Ttype. Including the temporal disambiguation module into Chrono increased the Type Accuracy from 0.65 to 0.82. This large increase puts Chrono on par with the other state-of-the-art systems, however, it does not exceed them with MSRA still holding the highest Type Accuracy of 0.89.

**Table 6:** End-2-End results for state-of-the-art systems, Chrono, and Chrono+TTD (ClinBioBert-Seq2Seq-Ttype) using span-based P, R, and F1 metrics.

**System**	**P**	**R**	**F1**	**Type**	**Value**	**Modifier**
Mayo	0.88	0.92	0.9	0.86	0.73	0.86
Vanderbilt	0.83	0.91	0.87	0.85	0.7	0.85
MSRA	0.88	0.95	0.91	0.89	0.72	0.89
Chrono	0.78	0.9	0.84	0.65	0.56	0.8
Chrono+TTD	0.78	0.9	0.84	0.82	0.57	0.77

One limitation of Chrono is that the FREQUENCY type parsing has not been fully implemented, and is limited to identifying known abbreviations for frequency expressions. This could be a factor in the poor performance of Chrono, thus, all systems were re-evaluated after removing the FREQUENCY temporal phrases from the results and gold standard using the same filtering script as mentioned previously. Table 7 shows that Chrono's Type Accuracy does indeed increase from 0.82 to 0.89 such that it is greater than the Mayo and Vanderbilt systems, but it is still second to MSRA at 0.91. This indicates that FREQUENCY phrases are a contributing factor; however, they are not the only factor as Chrono's Precision is reduced while the Recall is improved resulting in an unchanged F1 score of 0.84 while the F1 scores of all other systems were improved. Thus, while Chrono is now on par with state-of-the-art systems, it still has room for improvement.

**Table 7:** End-2-End results for state-of-the-art systems, Chrono, and Chrono+TTD (ClinBioBert-Seq2Seq-Ttype), with FREQUENCY temporal phrases removed, using span-based P, R, and F1 metrics.

**System**	**P**	**R**	**F1**	**Type**	**Value**	**Modifier**
Mayo	0.91	0.91	0.91	0.86	0.72	0.84
Vanderbilt	0.84	0.92	0.88	0.87	0.71	0.85
MSRA	0.89	0.96	0.93	0.91	0.71	0.90
Chrono	0.76	0.94	0.84	0.69	0.60	0.83
Chrono+TTD	0.75	0.94	0.84	0.89	0.62	0.80

## 4. Discussion

In this work we provided a detailed error analysis of the top systems participating in the 2012 i2b2 Challenge, and identified six main issues that impeded performance on the poorest performing files. Lexical issues contributed the least to the poor performance of the top i2b2 systems, while more complex errors involving the properties and normalized value of temporal expressions contributed the most. Lexical, as well as Gold Standard, errors are relatively straightforward to fix; however, DURATION vs. DATE, Anchor Time, and Delta Value errors are more complex as they require context to understand and may not be able to be resolved with rules and regular expressions. The biggest problem all three systems had was resolving relative temporal expressions, such as “over the past 2 weeks”, “2 weeks prior” or “a few days later”. Determining whether these are DURATION or DATE types is the first challenge, then once a DATE type is assigned the system has to figure out the Anchor Time and Delta Value needed to calculate the correct date for a given relative temporal phrase. Both of these tasks can be complex as they both require knowledge of the context and some reasoning ability in order to correctly assign a date value. However, even with context, the temporal disambiguation task can be challenging for human annotators to agree on a classification depending on how they define an associated event, which can limit our ability to train an automated classifier. For example, in the sentence “...she was in her normal state of health until 3 days ago.” the gold annotates the phrase “3 days ago” as a DATE type, however, one could also view this as a DURATION of 3 days being in an abnormal state of health. These types of phrases are exceedingly difficult to classify as they require extensive use of context and domain knowledge; however, both could be correct as they would lead to a correct timeline reconstruction. This can be mitigated by strictly defining how DATE and DURATION are annotated in a gold standard corpus; however, this would limit an applications generalizability to other corpora or tasks. Future work in developing fuzzy evaluation metrics for these types of phrases could provide more realistic performance evaluations for the TERN task, lead to more generalizable tools, as well as mitigate the perpetuation of errors in upstream tasks such as timeline extraction.

While Chrono performed on par with the top i2b2 systems with respect to Recall, it was clear through the error analysis that it was not as mature. Chrono was unable to correctly parse out parameters and determine normalized values of complex and relative temporal expressions, which are difficult to normalize because their value always depends on another temporal expression or some event that is either implicit knowledge or information located in another part of the document. However, before they can be normalized to a value and be placed on a timeline, their temporal type must be determined. To address this, we implemented a temporal disambiguation module in Chrono to disambiguate Period and Calendar-Interval SCATE types as either a DATE or DURATION type. This module addresses the first step toward improving the normalization of relative temporal expressions: the disambiguation and correct classification of DATE and DURATION temporal types.

To implement a temporal disambiguation module in Chrono, we utilized contextualized embeddings from temporally fine-tuned BERT models. We found that incorporating the contextualized word embeddings into classical learning models, such as SVMs, reaches state-of-the-art performance for the DATE/DURATION temporal disambiguation task on the RelIV-TIMEX corpus. Additionally, temporally fine-tuning BERT models on complex tasks (e.g., Seq2Seq) create contextualized word embeddings that increase the performance of classification models on the disambiguation task. Finally, while adding context generally degrades performance, this feature extraction strategy can help unmodified BertBase embeddings compensate for domain shifts. These results show that utilizing temporally fine-tuned contextualized word embeddings can improve system performance on the TERN task, and leads to the question of whether this strategy could be utilized for other temporal reasoning tasks.

## 5. Limitations

While Chrono does achieve the highest F1 scores on the RelIV-TIMEX data set, it is by a slim margin with confidence intervals that overlap the top i2b2 systems (Supplementary Table 6); thus, there are several limitations one should take into consideration when interpreting the results of this work. First, the error analysis was performed using the results generated by the top three i2b2 systems on the 2012 i2b2 test corpus, which is the same data set used in Chrono's end to end evaluation. This was done because we did not have access to the results run on the training corpus due to some of the tools not being available. This could create bias in the results, however, the error analysis on the test data set was only used to identify the task that was difficult for these systems (i.e., relative temporal type disambiguation) and none of the test data or individual errors identified in the error analysis were utilized in algorithm improvements, the fine-tuning tasks, or the training of the disambiguation module, which should mitigate egregious bias in the results. Also, the initial algorithm improvements discussed in Sections 2.5, 3.1 were done using the training data set and then evaluated in Figure 2 using the same training data set, so performance should be interpreted with this in mind. Additionally, when implementing a method to summarize multiple subword embeddings to represent an entire whitespace tokenized token we chose to only use the last subword embedding. There are other ways to implement this, including taking the average or sum over all subwords, however, their effectiveness has not been formally assessed. Evaluating the effects of these various methods within Chrono and other applications is being explored. Finally, when looking at the absolute counts of errors for Chrono and the other systems (Supplementary Tables 1, 2) it is clear Chrono still has more errors than the top i2b2 systems; however, some of this is due to Chrono annotating temporal expressions, such as age-based expressions, that are not included in the gold standard or the top systems. In addition, as mentioned previously, Chrono still requires additional work in other areas such as annotating FREQUENCY and calculating the correct normalized value of temporal expressions, both of which contribute to the inflated error count.

## 6. Conclusions and future work

In conclusion, this work has made progress in the area of temporal recognition and normalization by (1) identifying specific areas in need of improvement through a detailed error analysis of the top performing i2b2 systems, (2) showing that temporal information can be infused in contextualized embeddings extracted from BERT models, (3) improving the ability of systems to disambiguate DATE and DURATION relative temporal phrases without the need to develop a complex rule-base, and 4) providing the first dual-parsing TERN system, Chrono, that normalizes temporal expressions into both the SCATE and ISO-TimeML schemes. Future work will include improving the classification of relative temporal expressions that require a deeper understanding of semantics, such as the type of event. The next step beyond temporal type disambiguation will be to identify an anchor time and delta value so that relative temporal expressions can be correctly normalized. Finally, investigation of whether ensemble classifiers and other learning models utilizing temporally fine-tuned contextualized embeddings can contribute to the TERN task should be explored.

## Data availability statement

The gold standard RelIV-TIMEX and DD-TIMEX data sets generated for this study have been deposited in the [DBMI Data Portal] [https://portal.dbmi.hms.harvard.edu/]. Fine-tuned BERT models can be downloaded from https://bit.ly/temporal-bert. Chrono is available on GitHub at https://github.com/AmyOlex/Chrono. The error analysis and gold standard filtering scripts used in this work are also available on GitHub at https://github.com/OlexLab/i2b2_bootstrap_error_analysis and https://github.com/OlexLab/gold-standard-utils, respectively.

## Author contributions

AO led experimental design, implementation, all analyses, and drafted the initial version of this manuscript. BM oversaw all aspects of this work and edited drafts of this manuscript. Both authors read and approved the final version of this manuscript.

## Funding

AO was supported in part by CTSA award no. UL1TR002649 from the National Center for Advancing Translational Sciences. Its contents are solely the responsibility of the authors and do not necessarily represent official views of the National Center for Advancing Translational Sciences or the National Institutes of Health.

## Conflict of interest

The authors declare that the research was conducted in the absence of any commercial or financial relationships that could be construed as a potential conflict of interest.

## Publisher's note

All claims expressed in this article are solely those of the authors and do not necessarily represent those of their affiliated organizations, or those of the publisher, the editors and the reviewers. Any product that may be evaluated in this article, or claim that may be made by its manufacturer, is not guaranteed or endorsed by the publisher.

## Footnotes

^1^ https://github.com/AmyOlex/Chrono

^2^Available at: https://github.com/OlexLab/i2b2_bootstrap_error_analysis.

^3^ https://www.powerscore.com/lsat/help/lsat-quantity-terminology.cfm

^4^Temporally fine-tuned BERT models are available for download at: https://bit.ly/temporal-bert.

^5^ https://mccormickml.com/2019/05/14/BERT-word-embeddings-tutorial/

^6^Script available at https://github.com/OlexLab/gold-standard-utils/blob/main/i2b2_ISO-TimeML/Filter_i2b2_XML.py.
