# Supplementary material for: Temporal disambiguation of relative temporal expressions in clinical texts
Source: Front Res Metr Anal. 2022 Oct 24;7:1001266. doi: 10.3389/frma.2022.1001266 (PMC9638055; doi:10.3389/frma.2022.1001266)
Supplement: Supplementary file 1 [file Data_Sheet_1.pdf]

Supplementary Material

1 SUPPLEMENTARY FILE 1: COMPATIBILITY OF SCATE AND TIMEML ANNOTATION SCHEMES

To evaluate Chrono on the i2b2 data using the i2b2 scripts, the SCATE annotations needed to be converted to TimeML. While SCATE contains enough information to be converted to TimeML, the TimeML annotations do not contain enough information to be effectively converted to SCATE entities. This is due to the saved annotations in TimeML being normalized into the ISO standard. For example, the phrase “Thursday, June 3, 2000 at 12pm” would be saved in ISO as “06-03-2000T12:00:00”. This representation does not annotate the day-of-week mention “Thursday”, but SCATE does (even though it is redundant), and it is not clear from the ISO format if the text contains an AMPM entity, and second-of-minute, or a minute-of-hour entity, all of which must be annotated by SCATE if present to be counted as correct. Additionally, SCATE differentiates “Periods” and “Calendar Intervals” whereas TimeML treats them both as a DURATION or DATE, and it would not be straightforward to differentiate between them when converting to SCATE. For example, TimeML would annotated the token “week” as a DURATION in the following two phrases: “I have had pain for the past week.”, “I had pain all last week”. However, SCATE would annotated the first as a Period and the second as a Calendar Interval. The main different between these schemes is that SCATE is focused on the intervals of time while TimeML is focused on if the event associated with the interval happened continuously throughout said interval or only occurred at a specific point in time at the beginning or end of the interval mentioned in the text. This makes it difficult to convert based solely on either the TimeML or SCATE annotations, so additional measures need to be taken to include phrase context when converting these entities from SCATE to TimeML.

Converting SCATE to TimeML is possible as the SCATE data can be distilled down into the ISO format for DATE types and many DURATIONs as well. However, there are still challenges in retrieving a good conversion, and any conversion script would still need access to the full text document. Thus, it was decided to integrate the needed TimeML information into the existing SCATE objects within Chrono and provide an additional input/output mode for TimeML annotations instead of building a stand-alone conversion script.

2 SUPPLEMENTARY FILE 2: CHRONO MODIFICATIONS

2.1 Conversion Changes

ISO Formatting: The first change to Chrono was to convert explicit date/time strings to ISO format and store the normalized value for each temporal expression. This was done using an existing 3rd-party ISO conversion module in python named dateutil. Initially, the raw temporal phrase identified by Chrono was input into this tool; however, some raw phrases were not able to be parsed by dateutil, such as phrases that are part of the document metadata or header lines. Thus, for ISO conversion, a string was re-generated from the SCATE entities associated with a temporal phrase to be passed into the ISO conversion module. An example of a raw phrase that dateutil can not parse, but Chrono can is show in Quotation 1.

911203 Tuesday December 4A 1991 WEST (1)

Other phrases the dateutil method cannot handle are fuzzy and referential phrases such as “yesterday” or “3 days ago”. In addition, phrases such as “last Saturday at 3pm” were also parsed incorrectly as the “last” was ignored and the normalized ISO string would reference the next Saturday. For this later issue, proper setting of the reference time is required prior to conversion. For the former issue, a more complex solution is needed for proper normalization.

Finally, for proper ISO conversion of 2-digit years, such as ’97’, that are not part of date strings, a proper reference time had to be set in the dateutil method. This is simply set as the document creation time.

Periods and Intervals: Periods and Calendar Intervals are converted to DURATION ISO notation as this is the most frequent classification of these entities. This format must include the designation for a period (P), the number associated with the durations, and the units of the duration (e.g. D=days, M=months, Y=years, W=weeks). If the units are in seconds, minutes, or hours, the period designation must be accompanied by a “T”. For example, “3 months” would be coded as “P3M”, and the phrase “3 minutes” would be “PT3M”. Durations representing the same length of time, such as P1D and PT24H, are considered equivalent.

SCATE entities are clearly labeled as being a Period or Calendar-Interval, where both are primarily coded as a DURATION in TimeML. If a number is associated with a SCATE entity, this is easily retrievable. Thus, implementing this conversion was straightforward for the majority of SCATE Period and Calendar-Interval entities; however, some SCATE Periods and Calendar-Intervals are actually annotated as a DATE in TimeML. Developing rules for this differentiation is difficult, so we decided to set all to DURATION at this point in time. The impact of this decision on performance is discussed in the error analysis section of the main paper.

Approximate Phrases: Another conversion from SCATE to TimeML was the conversion of entities with approximate modifiers. These entities required the TimeML APPROX attribute to be set along with a DURATION that had a number associated with it. While SCATE annotates these modifiers, it does not associate a number with them. Approximate phrases included “several days”, “several minutes”, “many days”, etc. These have an annotation in ISO such as “P3D”. However, choosing a number for the terms “several”, “many”, “few”, etc. is challenging as the exact duration may be interpreted differently depending on the reader, context, and the magnitude of the units involved. For example, “a few minutes” could mean around 5 to 10 minutes, while “a few months” is more likely to be around 2 to 3. An analysis of these types of phrases was performed to determine what the consensus was in the i2b2 gold standard training data set to inform the development of rules to convert these expressions from SCATE to TimeML.

An analysis of temporal expressions having the “APPROX” attribute set in the i2b2 gold standard revealed inconsistencies as to the exact numerical value with which these phrases were annotated. This was especially true for the modifier words “several” and “few”. Numbers associated with “several” include 2, 3, 4, and 5. Numbers associated with “few” include 2, 3, and 4. Numbers associated with “many” include 5, 10 and 30. Even the same temporal expression was annotated with different values. For example, “several days” is coded as “P3D” in one document, and “P4D” in another. Similarly, “many days” is coded as P10D in one document, and P30D in another. This inconsistency makes it very difficult to correctly annotated these phrases and may impact performance.

In Chrono, these approximate modifiers were set to be a consistent value based on the average gold standard annotations and the descriptions for these terms on the LSAT exam[1]. In Chrono, the terms “few” and “several” are set to “3”, and “many” is set to “10”. Plural time expression, like “weeks”, without a

[1] https://www.powerscore.com/lsat/help/lsat-quantity-terminology.cfm

number or approximate modifier are set to “2” with a modifiers of “NA”, and any singular period or interval is defaulted to a value of “1” with a modifier of “NA”.

2.2 Algorithm Improvements

The next round of improvements made to Chrono were focused on improving the Recall. The Precision numbers will naturally be lower because Chrono was built to identify a wider range of temporal expressions than what was annotated in i2b2. Thus, we focused on improving recall first, followed by the Value Accuracy.

Clinical Abbreviations: The i2b2 training data set contained a number of clinical abbreviations that actually represented a frequency. For example, “bid” represents “twice a day”. To account for this, a new dictionary was created that contained a large list of temporal abbreviations used in clinical settings[2]. All of these abbreviations represented frequencies, thus, a new Frequency method was created to parse these phrases. Currently, only the abbreviations are parsed as frequencies, and none of the properties are being set. Future work will require setting the properties correctly and identifying frequencies that don’t include abbreviations.

2-Place Dates: Two-place dates are tricky. They can either be of the format MM/YY, M/YY, MM/YYYY, MM/DD, or M/DD. If a 4-digit year is found, then it is unambiguous as to which place is a day, month, and year. However, if a 2-digit year is present, or the format is NN/NN or N/NN, it is unclear as to which place refers to day, month, and year.

Initially, Chrono was not recognizing 2-place dates at all as it looked for the standard 3-place format. Upon editing the code to identify 2-place dates as well, the issue became differentiating dates from test results and the formats MM/YY, M/YY, MM/DD, and M/DD. Chrono deals with this issue by constraining 2-place dates to have specific ranges of values. In a string with the format XX/NN or X/NN, the X or XX must be a numerical value between 01 and 12. If it is not the string is considered to not be a date. If the first place is determined to fit in the range for a month, the then second place must be between 1 and 31 to be classified as a day. If the second place is greater than 31 then it is classified as a 2-digit year. Now, this will of course run into situations where these rules will prohibit the date value from being interpreted correctly. For example, the string “01/10” could mean January 2010 or January 10th. Chrono would assign the later value to this string. While this may seem like a large issue, usually, when dates between 1/1/2000 and beyond are now relayed, the full 4-digit year is written for clarity, so we expect to not have too many issues with these rules. However, future work could include a machine learning algorithm to use the context of the passage to determine if the last 2 digits are representing a year or a day.

3 SUPPLEMENTARY FILE 3: CHRONO’S PERFORMANCE AFTER EACH MODIFICATION

3.1 Out-of-the-box Performance: After Conversion Changes

After updating Chrono to output SCATE annotations into TimeML format we assessed it’s “out-of-the-box” performance. The i2b2 evaluation script was run to generate the aggregate performance of Chrono annotations for only TIMEXs and using overlapping span. Overlapping spans was chosen as Chrono spans are not directly coded to i2b2 standards; thus, it is enough to know our spans overlap, which means we annotated approximately the correct phrase. After Conversion Modifications, the overall Precision was

[2] https://en.wikipedia.org/wiki/List_of_medical_abbreviations:_B

0.56, Recall 0.81, and F1 0.66, which (except for Precision) are better than even the improved performance of Chrono on the THYME corpus Olex et al. (2019).

Performing an error analysis of Chrono's performance on the training data set revealed that the low Precision was due to Chrono annotating a lot of relative temporal and age-related expressions that i2b2 did not. For example, the term "recent" in the phrase "...go but not on home 02 with recent FEV1 27% of predicted value", and the term "now" in the phrase "...kidney transplantation and now has good graft function" provide ordering information in the clinical note for events, but are not annotated by i2b2 because they cannot be directly linked to a frequency, duration, date, or time. Age-related phrases include "28-year-old" and "72 years", which are both specifically annotated in SCATE but not in i2b2. Chrono was also missing many temporal clinical abbreviations, such as "bid", and was unable to parse 2-place dates formatted as "MM/YY" or "DD/MM". Additionally, Chrono missed phrases like "postoperative day 2". As Chrono is primarily rule-based, these stylistic writing differences between the THYME data set and the i2b2 clinical notes were not coded. Thus, further improvements to Chrono were made to account for these additional elements in the Algorithm Improvements phase.

Additional sources of error include differentiating DURATION and DATE types. Chrono is coded to convert all SCATE Periods and Calendar Intervals to DURATION types in TimeML. However, some of these mentions are actually annotated as DATE types in the gold standard. For example, in the phrase "One week prior to presentation , he had chest pain.." the temporal expression "One week prior" is coded by Chrono as a DURATION, but in the gold annotations it is a DATE that is set to the day one week prior to the admission date. Similarly, phrases such as "On postoperative day #4" are annotated as a DURATION of 1 day by Chrono, but are given a specific date in the gold annotations. Both of these phrases require an anchor time and interval delta from the anchor time in order to calculate the date accurately. At the time, these errors were few compared to the lexical issues mentioned previously. Thus, these errors were not addressed in the next round of Chrono modifications, which was focused on improving recall performance.

3.2 Improved Performance: After Algorithm Improvements

Upon implementation of identifying clinical abbreviations and 2-place dates, Chrono's performance on the i2b2 training data set increased to a Precision of 0.66, Recall of 0.92, and F1 value of 0.77 (main text Figure 2, Light Purple). Property attributes for Type, Value, and Modifier also increased, but are still below those of the state-of-the-art systems submitted to the 2012 i2b2 Challenge. Running Chrono for the first time on the unseen Evaluation data set from the i2b2 challenge resulted in similar performance to the improved training run with only a slight drop in Precision, Recall, and F1 (main text Figure 2, Light Green).

Even in the Evaluation corpus we see that Precision is low, and pulls the F1 value down due to Chrono annotating additional types of temporal tokens not annotated by i2b2. In order to assess how much these tokens were affecting the Precision, we implemented a toggle in Chrono to turn off the annotation of relative and vague temporal tokens such as "briefly" and "recently". Figure 2 in the main text shows the changed results with this toggle turned on. As can be seen, removing these relative terms increased the Precision and F1 measure without affecting the Recall or other properties (main text Figure 2, Green), which affirms these extra terms were the issue.

4 SUPPLEMENTARY FILE 4

4.1 Additional LEXICAL Examples

- All three systems missed the phrases “three cycles” and the token “one” in “one dose”, both of which were annotated as a FREQUENCY in gold.
- Mayo and Vanderbilt (the rule-base systems) only annotated the “day” token in the phrase “day +11” where the hybrid system captured the full phrase.
- The rule-based systems also missed annotating the phrases “3 / week”, “14d”, and “2 wk”, but the hybrid MSRA system did capture all these phrase; however, it did not assign the correct type to any of them.
- Mayo and Vanderbilt only annotated “weeks” in the phrase “one and a half weeks”, and MSRA missed annotating the token “later” in the phrase “A few days later” where the two rule-based systems captured the full phrase.
- MRSA was the only one of the three to consistently annotate the token “sat” in phrases like “and o2 sat stable” as a DATE when it was actually referring to oxygen saturation.

4.2 Additional FREQUENCY Examples

- The rule-based systems seem to prioritize a FREQUENCY annotation over DURATION both in the examples in the main text and with the phrase “times one month”. Both Mayo and Vanderbilt only annotated the tokens “times one” and missed the “month” leading to this phrase being incorrectly annotated as a FREQUENCY when it should have been a DURATION. The MSRA system correctly captured the entire phrase “times one month” and gave it the correct DURATION temporal type.
- All three systems had trouble with the phrases “with a Vision stent , 3 x 18”, “negative CK X4”, and “negative troponin X4” that were all from the same file. All systems annotated the tokens “x 18” and “X4” as FREQUENCY types when they were not included in the gold standard as a temporal phrase.

4.3 Additional Discussion on DURATION vs DATE Errors

Three phrases (1, 2, and 3 in Figure 4 of the main text) were incorrectly classified by all systems. In the SCATE schema, each of these three phrases would be listed as a Period type, however, in the TimeML schema two are DATE types (phrases 1 and 2) and one is DURATION (phrase 3). The key difference is that the two DATE type phrases are referring to a discrete event that will happen in one year (a CT scan) or one month (an ultrasound), whereas the DURATION phrase is referring to an event that has continuously happened over the course of three days (black stools). Phrase 3 is probably among the most difficult for any system to parse because it requires prior knowledge that the event of “black stools” is not discrete and can occur over multiple days.

Several phrases were consistently classified incorrectly by the Mayo and Vanderbilt systems. These include phrases 4, 5, and 6 in Figure 4 of the main text, which all reference dates in the past as indicated by the word “prior”. Each of the rule-based systems seemed to miss this key word and assign these phrases to the DURATION type when they should have been a DATE. The hybrid MSRA system classified these instances correctly as a DATE; however, another instance of the word “prior” appears in phrase 7 and was classified correctly by Mayo as a DURATION, but incorrectly by Vanderbilt and MSRA as a DATE. Interestingly, phrase 8 also contains the token “prior” and is consistently classified incorrectly by Mayo as a DURATION, however, unlike the other phrases that include the word “prior”, Vanderbilt identified this one correctly as a DATE along with MSRA. This indicates that each system may have a rule dictating

priority over how these types of phrases are classified that potentially include key context words. Vanderbilt may have included the key word “until” in its rule-base, which may have led to the correct classification for this phrase.

The system from Mayo had particular trouble annotating the phrase “the day” that appeared twice in 2 files (phrases 9 and 10 were from one note and phrases 11 and 12 were from another). In each instance the phrase “the day” was annotated as a DATE by the gold standard, however, Mayo marked these as DURATION types while Vanderbilt and MSRA correctly classified them as DATE. Note the context for each instance of the same phrase “the day” is different for each occurrence. This actually leads to the values being different for each, however that is related to anchor time issues and will be discussed subsequently. A third file also contained the phrase “the day” (phrase 13), but gold annotated “the day PTA”, which means “the day prior to admission”. Again, Mayo defaulted to classifying this as a DURATION while Vanderbilt and MSRA correctly identified it as a DATE type.

Out of all 17 phrases, Mayo only got 3 correct (phrase 7, 14, and 15). As discussed above, Mayo most likely has a rule that classifies any phrase such as “two weeks” as a DURATION as it did this consistently regardless of the context. Interestingly, phrases 14 and 15 were both classified correctly by Mayo and incorrectly by MSRA. Both of these phrases include the key context word “later”, which was probably the signal word for a DATE classification in Mayo’s system and was not annotated as part of the phrase by MSRA. Vanderbilt also classified phrase 14 correctly and annotated the word “later” as part of the phrase, but got phrase 15 wrong as it missed annotating the key context word “later” indicating this was a DATE and not a DURATION. This may have been the result of Vanderbilt’s system using different rule sets to annotate these two phrases that handled the token “later” differently.

For the last two phrases listed in Figure 4 of the main text, phrases 16 and 17, neither Mayo nor Vanderbilt recognized these as temporal phrases. They were identified by MSRA as temporal, but the temporal type classification was wrong on both accounts. To normalize both of these phrases correctly, knowledge of clinical shorthand is required (e.g. “d/c” means discharge) and an understanding of the context and type of event (continuous or discrete) is needed. Even if MSRA used a machine learning module to classify temporal phrases as DATE or DURATION (note, it is unknown if they did), these two phrases would probably still present a challenge.

Finally, lets briefly revisit phrases 7 and 8, which are both from the same file, and both contain the same temporal phrase “two weeks prior”, but one is annotated as a DATE and the other as a DURATION. All three systems were consistent in annotating these phrases and thus, each got one right and one wrong. This indicates static rules may have been implemented that do not take all the context into account in order to classify these phrases correctly. The complex and sometimes subtle contextual clues that humans can pick up on easily are clearly demonstrated throughout all of the examples in Figure 4 of the main text where even the same temporal phrase can have a different meaning depending on the context (and as we will see in the next section can have different values as well). Thus, developing an exhaustive set of rules to identify any DURATION or DATE in any context is infeasible due to the variety of potential lexical and semantic forms; however, a machine learning model may be able to pull this off with the right features. While it is unknown if the MSRA system actually used a machine learning model for this task, it is clear that this system did perform better than either of the two rule-based systems on these difficult phrases.

4.4 Additional Discussion on Anchor Time and Delta Value Errors

Context Switching: Deciphering when the context switches from being written upon admission to being written on discharge was difficult for all systems. As discussed in the main text, phrases 1, 2, and 3 in Figure

5 from the main text relay some phrases that include the temporal words “yesterday” and “today” in the same file, however, one has the admission time as an anchor while to other refers to the date of discharge. All three systems calculated a different, and incorrect, date for the “yesterday” phrase. Vanderbilt seems to assume it was the day of admission so was off by 1 day. Mayo assigned a date of 11/16/2006, which seems to have come from a DATE annotation in the previous sentence with the phrase “...history of CAD status post non ST elevation MI in 11/17 who presents with chest pain...”. Mayo annotated the token “11/17” as a date when it was not annotated by gold. From this file, and many others, it seems the Mayo system uses the most recently annotated DATE as the anchor for many of these relative phrases. Similarly, the MSRA system may have similar logic as it annotated the phrase “Yesterday morning” as the year “2003”. Looking at the context, it seems to have gotten this from the prior phrase “In 2003 , he had..”. While Mayo and Vanderbilt annotated “2003” as a year, they did not consider it as an anchor date. For the “today” term, there are two phrases in this file (phrases 2 and 3), and gold gives the same value (the discharge date) to both of them. For phrase 2, Mayo is still using the 11/16/2006 date from the previous section as the anchor time, while Vanderbilt and MSRA assume “today” is referring to the admission date. Interestingly, for the second “today” phrase the context points directly to the day of discharge and MSRA was able to get the correct date; however, both Mayo and Vanderbilt have the date 6/18/2006. This seemingly came from a new DATE having been annotated in the context prior to this last phrase, “June 18 , 2006 , at 8:30 p.m.”, showing that both Mayo and Vanderbilt have rules for anchor times that depend on the last annotated DATE regardless of the rest of the context.

Deciphering Admission or Discharge as a Anchor: As discussed in the main text, one of the difficult files references the day of admission, the day prior to admission and a day 2 weeks after discharge (phrases 4 through 6). Mayo actually fails to annotated 2 of these 3 phrases as a DATE to start with and has them listed as DURATION types. For phrase 4, MSRA is the closest, but misses the key word “prior” and ends up assigning this phrase as the day of admission when it should have been the day prior to admission. Vanderbilt seems to be using the last annotated date from several sentences prior in the phrase “...was weaned off her pressors on 02-21...” as the anchor date as it doesn’t recognize the term “call-out” to indicate the day of admission. Both Vanderbilt and MSRA also use this same date for the next 2 phrases (5 and 6) “the day” and “at this time”, both of which require knowledge of the context to calculate correctly. Phrase 5 should be more straightforward with the immediate context. Instead of “the day prior to call-out” from phrase 4, we have a shift in context for phrase 13 with “the day of call-out”. Phrase 6 requires context from further away and over multiple sentences. The full phrase is “...was followed by urology during her stay and will see them again 2 wk after d/c...At this time , urology will coordinate removal of...”. Note the phrase “will see them again 2 wk after d/c” that refers to a date 2 weeks after discharge. This requires the parsing of the token “d/c”, which none of the systems seem able to do, and the knowledge of they will be seeing “urology” again and “urology” will be the one to coordinate a procedure, so this time it would be correct to use the previous date obtained from “2 wk after d/c” for the phrase “At this time”. Since none of the systems classified “2 wk” as a DATE, they didn’t have that information to go off of. If they did then the rules shown previously about using the last annotated date would probably have led to obtaining the correct anchor time in this instance; however, that doesn’t always work. For example, in phrase 14 “...underwent cardiac catheterization today...”, the term “today” was annotated by all 3 systems, but the date was calculated incorrectly, because all 3 systems used some other previously annotated date as the anchor instead of setting “today” as the date of admission, which is what was provided by the gold standard. Thus, having a blanket rule to classify these referential dates as the last annotated will certainly catch some, but will not be very precise.

Finally, two additional phrases demonstrate the difficulty of figuring out whether to use the admission date, discharge date, or some other date from the context. Phrase 15 provides an example similar to phrase 6 where the anchor date is another relative phrase in the context prior. The full phrase plus context is "...until one and a half weeks prior to admission ... was prescribed cortisone drops . A few days later she complained of dizziness ." Before being able to identify the date for "A few days later" you first have to identify the date for the phrase "one and a half weeks prior to admission". In this instance, the "prior to admission" should be a straightforward clue as to what the anchor date is for this phrase, but one would need to be able to link it to the following phrase "A few days later". The MSRA system annotated "few days" as a DURATION, so did not provide a DATE, however, the Mayo and Vanderbilt systems did provide a date albeit the wrong one. The key was not being able to annotated the "one and a half weeks" phrase correctly, so Mayo chose to use the discharge date as the anchor, and Vanderbilt chose to the use admission date. Any phrase with the term "admission" or "discharge" seems like it would be simple to parse. Some systems seemed to utilize these keywords while others did not. For example, another phrase shown in Supplementary Table S4 below "...with chronic mild dyspnea on exertion until two weeks prior to admission ." Mayo annotated "2 weeks" as a DURATION, Vanderbilt correctly chose the admission date as the anchor, but MSRA chose the discharge date as the anchor, so calculated the wrong date.

Multiple days of care: An interesting, but problematic, file included several "postoperative day number X" phrases followed by "at this time" phrases (Supplementary Table S4, File 73). For this file, it was important to keep track of the most recently annotated date as the narrative was describing the events after a surgery event. The systems seemed to be able to do this, however, choosing the correct anchor time was difficult for Mayo and identifying the delta value was a challenge for MSRA. The Vanderbilt system was able to calculate the correct dates for all instances in this particular file. For this particular file, the admission date was 8/17/2009. One may assume that a surgery would have been performed on the day of admission in most cases, and this is exactly what the Mayo system does. Mayo was able to correctly identify the delta values to calculate the remaining "postoperative day" phrases; however, because this system assumed the anchor time was the day of admission the values were consistently off by a few days. In actuality, the key phrase "the patient was taken to the Operating Room on 2009-08-24" should have set the anchor time for all the postop phrases. Vanderbilt was able to identify this correctly, and thus obtained all correct dates that matched the gold annotations. MSRA was also able to ascertain this anchor date; however, this system was unable to process the delta value correctly when they were spelled out, which resulted in most of the postop phrases being set to the day of the surgery. This conclusion was reached because MSRA was able to obtain the correct calculated "postoperative day number 17" when a number was used instead of a word. In addition, all systems were able to assign the "correct" values to the various "at this time" phrases, as these phrase values match the postop day date assigned in the previous sentence. The performance of each system on this file indicates the importance of not just assuming an operation or other medical event happened on the day of admission and instead looking for contextual clues as to what the anchor time should be for each phrase.

First Days of Life: Similar to the "postoperative day number X" phrases in the previous paragraph, another file described the care of a newborn throughout the first month of its life referring to days of birth as "day of life X" (Supplementary Table S4, File 142). Similar to the issues MSRA had above with not recognizing any delta values that were spelled out, it assigned all values to be the day of admission. Both Mayo and Vanderbilt did perform calculations, however, they were off from the gold standard consistently by 1 day. Further investigation revealed that these two systems were using the day of admission as the first day of life; however, gold says the first day of life was the day after admission. This is a bit difficult for even a person to decipher because of phrase 8 "Mother presented on *day* of delivery with preterm labor...",

which would indicate that the first day of life may be the admission date. However, further reading reveals the context of phrase 10 includes a specified date: "...was discontinued on 05-23 (day of life 18)." Using this information to back-calculate when day of life 1 was we end up with the anchor date of 5/6/2016 instead of the admission date 5/5/2016. Notably, identifying this particular anchor date is a very complex task and requires high-level reasoning. Thus, identifying a single algorithm or machine learning model to calculate this will be challenging if possible at all. The Mayo and Vanderbilt systems were only a day off and had all the delta values correct, so this doesn't seem too bad; however, in the previous file discussing postoperative event the operation event was more than a day away from the admission date, so it is not always good to assume the admission date is the anchor date. Assuming the day of delivery is the admission date probably does catch many of these types of files, for example, phrase 11 in Table S4 is from another file and references "day of life three". Both Mayo and Vanderbilt get it correct by assume the admission date was the day of delivery, and MSRA has the now familiar problem of assigning this phrase the anchor time (admission date) because it can't parse out "three" as a delta value.

SUPPLEMENTARY TABLES AND FIGURES

System	Total Errors	Value Error Total	Value Error Rel DATE/DUR	Label Error Total	Label Error Rel DATE/DUR	Total Missed	Total Added	TIME			DATE			DURATION			FREQUENCY		
								Mislabeled	Missed	Added	Mislabeled	Missed	Added	Mislabeled	Missed	Added	Mislabeled	Missed	Added
Mayo	752	293	203	134	51	198	183	2	4	2	28	67	96	53	31	23	51	25	77
MSRA	744	350	297	109	64	219	168	5	5	5	57	21	150	40	13	16	7	27	48
Vanderbilt	885	301	223	120	51	297	171	0	7	1	71	67	212	34	45	27	15	48	57
Chrono (no TTD)	1313	157	89	574	372	420	893	53	9	52	50	52	79	471	16	289	0	85	0
Chrono (with TTD)	1254	527	385	145	43	421	177	11	9	59	51	52	316	76	15	38	7	85	8

Table S1. Count of total errors per category across all files. Column descriptions: Total Errors, count of any error type; Value Error Total, count of incorrect values for all annotations with a correct label; Value Error Rel DATE/DUR, subset of all value errors for relative DATE or DURATION expressions; Label Error Total, count of incorrect label annotations; Label Error Rel DATE/DUR, subset of all label errors for relative DATE or DURATION expressions; Total Missed, count of expressions annotated in the gold standard and not annotated by the system; Total Added, count of expressions annotated by the system and not present in the gold standard; TIME/DATE/DURATION/FREQUENCY-Mislabeled/Missed/Added, count divided by each temporal expression type for label errors and missed or added expressions.

System	Total Errors	Value Error Total	Value Error Rel DATE/DUR	Label Error Total	Label Error Rel DATE/DUR	Total Missed	Total Added	TIME			DATE			DURATION			FREQUENCY		
								Mislabeled	Missed	Added	Mislabeled	Missed	Added	Mislabeled	Missed	Added	Mislabeled	Missed	Added
Mayo	188	67	57	35	18	31	55	0	0	0	7	22	33	16	4	4	12	5	18
MSRA	155	71	63	20	13	11	53	2	0	1	7	7	38	11	0	2	0	4	12
Vanderbilt	192	74	60	27	13	27	64	0	1	0	13	19	44	9	1	4	5	6	16
Chrono (no TTD)	228	22	12	96	73	28	82	2	1	11	10	16	27	84	6	44	0	5	0
Chrono (with TTD)	213	84	67	19	10	28	82	0	1	11	6	16	64	13	6	7	0	5	0

Table S2. Count of total errors per category across 18 poorest performing files. See legend of Table S1 for column descriptions.

System	Value Error Total %	Value Error Rel DATE/DUR %	Label Error Total %	Label Error Rel DATE/DUR %	Total % Missed	Total % Added
Mayo	35.6	85.1	18.6	51.4	16.5	29.3
MSRA	45.8	88.7	12.9	65.0	7.1	34.2
Vanderbilt	38.5	81.1	14.1	48.1	14.1	33.3
Chrono (no TTD)	9.6	54.6	42.1	76.0	12.3	36.0
Chrono (with TTD)	39.4	79.8	8.9	52.6	13.1	38.5

Table S3. Percent of errors for value, label, missed, and added error categories out of all errors in the 18 poorest performing files for each system.

Main Text Phrase ID	File	Phrase	Context	Gold Value	Admission Date	Discharge Date	Mayo Value	Vanderbilt Value	MSRA Value
1	32	Yesterday morning	Yesterday morning , he developed...	5/12/2006	5/13/2006	5/16/2006	11/16/2006	5/13/2006	6/25/1905
2	32	today	On physical exam today	5/16/2006	5/13/2006	5/16/2006	11/16/2006	5/13/2006	5/13/2006
3	32	today	Prior to discharge today	5/16/2006	5/13/2006	5/16/2006	6/18/2006	6/18/2006	5/16/2006
7	73	postoperative day number two	Cholangiogram on postoperative day number two showed...	8/26/2009	8/17/2009	9/10/2009	8/19/2009	8/26/2009	8/24/2009
8	73	At the time	Cholangiogram on postoperative day number two...At the time...	8/26/2009	8/17/2009	9/10/2009	-	8/26/2009	8/24/2009
	73	the same time	Cholangiogram on postoperative day number two...At the time...At the same time	8/26/2009	8/17/2009	9/10/2009	8/19/2009	-	8/24/2009
	73	postoperative day number eight	On postoperative day number eight...	9/1/2009	8/17/2009	9/10/2009	8/25/2009	9/1/2009	8/24/2009
	73	at the time	On postoperative day number eight...Chest x-ray and sputum culture obtained at the time...	9/1/2009	8/17/2009	9/10/2009	-	9/1/2009	-
	73	postoperative day number ten	On postoperative day number ten...	9/3/2009	8/17/2009	9/10/2009	8/27/2009	9/3/2009	8/24/2009
9	73	postoperative day number 17	On postoperative day number 17...	9/10/2009	8/17/2009	9/10/2009	9/3/2009	9/10/2009	9/10/2009
10	73	At the time	On postoperative day number 17...At the time...	9/10/2009	8/17/2009	9/10/2009	-	9/10/2009	9/10/2009
	137	1 year prior	...during his most recent admission 1 year prior .	10/10/2014	10/10/2015	10/21/2015	-	-	10/10/2015
	137	7 weeks prior to admission	...status-post gastric bypass ...7 weeks prior to admission who presented...	8/17/2015	10/10/2015	10/21/2015	-	-	2/7/2015
	208	1 week ago	...which had been discontinued about 1 week ago .	5/19/2018	5/26/2018	5/31/2018	5/1/2018	5/19/2018	4/17/2018
	233	the day	...started earlier in the day...	6/4/2015	6/4/2015	6/8/2015	-	6/8/2015	6/4/2015
	233	the day	...pain was intermittent through the day...	6/4/2015	6/4/2015	6/8/2015	-	6/8/2015	6/4/2015
	253	one week	...HSV outbreak occurred on 2017-09-13 approximately one week prior to delivery .	9/15/2017	9/22/2017	9/27/2017	-	-	9/8/2017
	253	day of life three	...serum bilirubin obtained on day of life three...	9/24/2017	9/22/2017	9/27/2017	9/24/2017	9/24/2017	9/22/2017
	253	day of life three	Antibiotics were discontinued on day of life three...	9/24/2017	9/22/2017	9/27/2017	9/24/2017	9/24/2017	9/22/2017
4	402	the day	...required a dilt gtt on the day prior to call-out...	2/17/2013	2/18/2013	2/27/2013	-	2/21/2013	2/18/2013
	402	this time	...was transitioned to PO diltiazem and has been in NSR since this time .	2/18/2013	2/18/2013	2/27/2013	-	2/21/2013	2/18/2013
5	402	the day	...transitioned to PO diltiazem on the day of call-out .	2/18/2013	2/18/2013	2/27/2013	-	2/21/2013	2/21/2013
6	402	At this time	...was followed by urology during her stay and will see them again 2 wk after d/c...At this time , urology will coordinate removal of...	3/13/2013	2/18/2013	2/27/2013	2/21/2013	2/21/2013	2/21/2013
	527	day 34/42	...and was discharged to rehab on day 34/42 of the vancomycin .	2/19/2017	2/4/2017	3/4/2017	3/9/2017	-	-
	527	day 11-24	...the plan was for steroid taper : 60 mg x 10 days (already completed) , 40 mg x 14 d (already completed) , 20 mg x 14 d (now day 11-24) , 10 mg x 10 d , 5 mg x 10 d .	3/4/2017	2/4/2017	3/4/2017	2/14/2017	11/24/2017	2/14/2017
	537	the day PTA	...daughter says that on the day PTA...	1/19/2014	1/20/2014	1/21/2014	-	2/3/2014	1/20/2014
14	737	today	...underwent cardiac catheterization today...	6/10/2015	6/10/2015	6/11/2015	5/4/2015	5/4/2015	9/2/2015
	737	now	He is now preop for...	6/10/2015	6/10/2015	6/11/2015	-	9/2/2015	-
15	767	A few days later	...until one and a half weeks prior to admission ... was prescribed cortisone drops . A few days later she complained of dizziness .	12/21/2009	12/28/2009	12/31/2009	1/3/2010	12/30/2009	-
	817	two weeks prior	...with chronic mild dyspnea on exertion until two weeks prior to admission .	4/6/2012	4/20/2012	5/1/2012	-	4/6/2012	4/19/2012
11	142	day	Mother presented on day of delivery with preterm labor...	5/5/2016	5/5/2016	6/15/2016	-	-	5/5/2016
12	142	day of life two	Infant was intubated on day of life two...	5/7/2016	5/5/2016	6/15/2016	5/6/2016	5/6/2016	5/5/2016
	142	day of life four	Infant was extubated on day of life four to CPAP...	5/9/2016	5/5/2016	6/15/2016	5/8/2016	5/8/2016	5/5/2016
	142	day of life six	...and transitioned to room air on day of life six .	5/11/2016	5/5/2016	6/15/2016	5/10/2016	5/10/2016	5/5/2016
	142	day of life six	Caffeine citrate was started on day of life six...	5/11/2016	5/5/2016	6/15/2016	5/10/2016	5/10/2016	5/5/2016
13	142	day of life 18	...was discontinued on 05-23 (day of life 18).	5/23/2016	5/5/2016	6/15/2016	5/22/2016	5/22/2016	5/5/2016
	142	day of life four	...started on enteral feedings on day of life four...	5/9/2016	5/5/2016	6/15/2016	5/8/2016	5/8/2016	5/5/2016
	142	day of life seven	...feedings of 150 cc per kilogram per day by day of life seven .	5/12/2016	5/5/2016	6/15/2016	5/11/2016	5/11/2016	5/5/2016
	142	day of life 11	...26 calories per ounce with ProMod by day of life 11 .	5/16/2016	5/5/2016	6/15/2016	5/15/2016	5/15/2016	5/5/2016
	142	day of life five	...most recent electrolytes on day of life five...	5/10/2016	5/5/2016	6/15/2016	5/9/2016	5/9/2016	5/5/2016
	142	day of life 25	On day of life 25 , calcium 10.1...	5/30/2016	5/5/2016	6/15/2016	5/29/2016	5/29/2016	5/5/2016
	142	day of life two	...started on double phototherapy on day of life two...	5/7/2016	5/5/2016	6/15/2016	5/6/2016	5/6/2016	5/5/2016
	142	day of life six	...was discontinued on day of life six .	5/11/2016	5/5/2016	6/15/2016	5/10/2016	5/10/2016	5/5/2016
	142	day of life seven	...bilirubin level on day of life seven was 5.3...	5/12/2016	5/5/2016	6/15/2016	5/11/2016	5/11/2016	5/5/2016
	142	day of life two	The most recent hematocrit on day of life two was 47% .	5/7/2016	5/5/2016	6/15/2016	5/6/2016	5/6/2016	5/5/2016
	142	day of life two	Repeat CBC on day of life two showed...	5/7/2016	5/5/2016	6/15/2016	5/6/2016	5/6/2016	5/5/2016
	142	day of life three	Repeat platelet count on day of life three was 229 .	5/8/2016	5/5/2016	6/15/2016	5/7/2016	5/7/2016	5/5/2016
	142	day of life seven	Head ultrasound on day of life seven showed...	5/12/2016	5/5/2016	6/15/2016	5/11/2016	5/11/2016	5/5/2016
	142	day of life 33	A repeat head ultrasound on day of life 33 06-07 showed...	6/7/2016	5/5/2016	6/15/2016	6/6/2016	6/6/2016	6/7/2016

Table S4. Expanded list of temporal phrases for which it was hard to correctly identify the Anchor Time and/or Delta Value. 'ID' column lists the phrase ID from Table 2 in the main text.

System	DATE	DURATION
Gold	429	307
Chrono	463	337
Mayo	455	335
Vanderbilt	458	337
MSRA	454	337

Table S5. Number of relative temporal phrases in state-of-the-art systems, Chrono, and the RelIV-TIMEX Gold Standard.

System	***Feature Strategy***	***Model***	***Precision 95% CI***	***Recall 95% CI***	***F1 95% CI***	***Accuracy 95% CI***
Chrono (no TTD)	-	-	(0.694, 0.774)	(0.413, 0.484)	(0.319, 0.404)	(0.452, 0.53)
Chrono + TTD	***Phrase Only***	BertBase *(baseline)*	(0.887, 0.93)	(0.805, 0.859)	(0.845, 0.891)	(0.887, 0.93)
Chrono + TTD	***Phrase Only***	BertBase-Binary	(0.887, 0.932)	(0.806, 0.859)	(0.846, 0.891)	(0.887, 0.931)
Chrono + TTD	***Phrase Only***	BertBase-Seq2Seq_Ttype	(0.904, 0.944)	(0.819, 0.872)	(0.859, 0.905)	(0.903, 0.944)
Chrono + TTD	***Phrase Only***	BertBase-Seq2Seq_BIO	(0.905, 0.946)	(0.821, 0.872)	(0.863, 0.906)	(0.905, 0.946)
Chrono + TTD	***Phrase Only***	ClinBioBert *(baseline)*	(0.907, 0.944)	(0.821, 0.872)	(0.864, 0.904)	(0.906, 0.944)
Chrono + TTD	***Phrase Only***	ClinBioBert-binary	(0.906, 0.944)	(0.82, 0.872)	(0.862, 0.905)	(0.905, 0.944)
Chrono + TTD	***Phrase Only***	ClinBioBert-Seq2Seq_Ttype	(0.916, 0.953)	(0.829, 0.88)	(0.871, 0.913)	(0.915, 0.952)
Chrono + TTD	***Phrase Only***	ClinBioBert-Seq2Seq_BIO	(0.904, 0.945)	(0.82, 0.874)	(0.861, 0.906)	(0.904, 0.944)
Chrono + TTD	***Phrase +Context***	BertBase	(0.911, 0.948)	(0.826, 0.875)	(0.867, 0.908)	(0.911, 0.948)
Chrono + TTD	***Phrase +Context***	ClinBioBERT	(0.902, 0.94)	(0.817, 0.867)	(0.859, 0.901)	(0.901, 0.94)
MSRA	-	-	(0.892, 0.933)	(0.844, 0.89)	(0.866, 0.909)	(0.89, 0.932)
Mayo	-	-	(0.889, 0.934)	(0.733, 0.793)	(0.805, 0.855)	(0.888, 0.933)
Vanderbilt	-	-	(0.909, 0.947)	(0.766, 0.821)	(0.832, 0.876)	(0.908, 0.945)

Table S6. Bootstrap analysis 95% confidence intervals for Phase 2 system evaluations. A bootstrap analysis was performed on all system results by sampling with replacement for 1000 iterations and calculating the mean and 95% confidence interval for each metric.

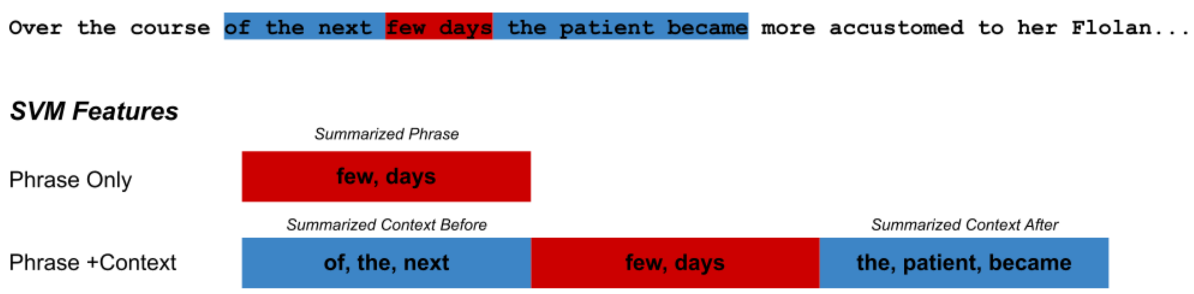

Figure S1. Overview of contextualized embedding feature extraction strategies. The example sentence highlights the temporal phrase (red) and context tokens with a window of 3 (blue). SVM feature components are summarized into a single embedding then concatenated as shown.

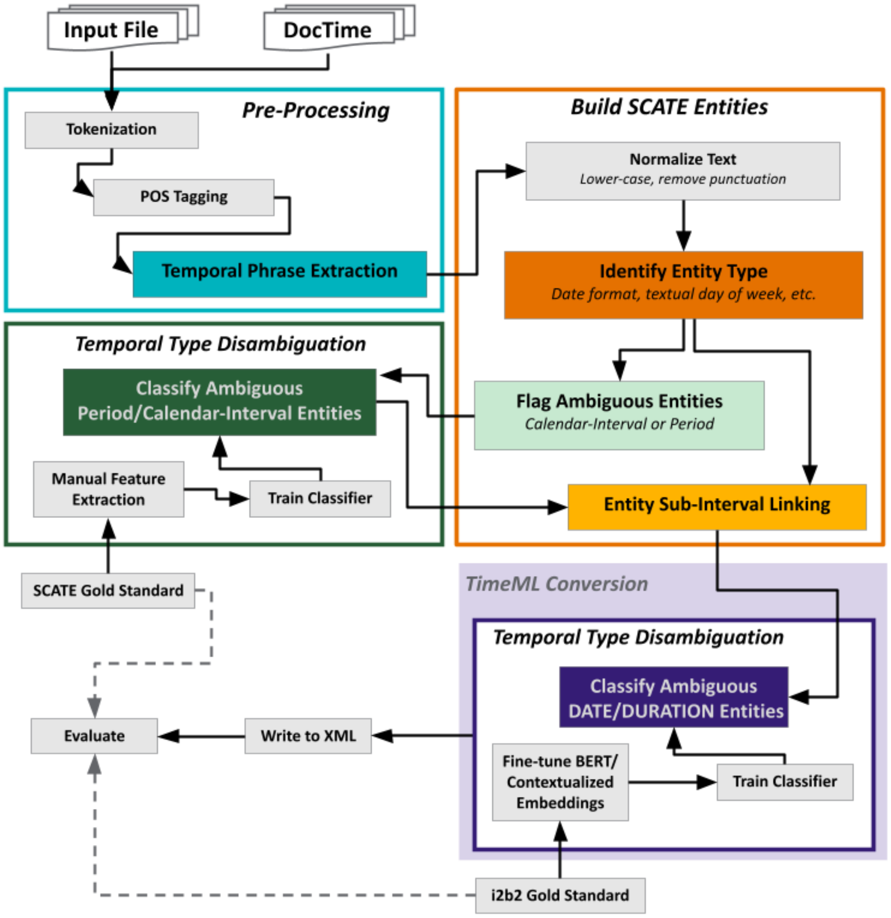

Figure S2. Chrono architecture with the DATE/DURATION Temporal Disambiguation Module.

REFERENCES

Olex, A., Maffey, L., and McInnes, B. (2019). NLP Whack-A-Mole: Challenges in Cross-Domain Temporal Expression Extraction. In *Proceedings of the 2019 Conference of the North American Chapter of the Association for Computational Linguistics: Human Language Technologies, Volume 1 (Long and Short Papers)* (Minneapolis, Minnesota: Association for Computational Linguistics), 3682–3692. doi:10.18653/v1/N19-1369
